# Identifying strategies that support equitable person-centred osteoarthritis care for diverse women: content analysis of guidelines

**DOI:** 10.1186/s12891-023-06877-x

**Published:** 2023-09-14

**Authors:** Chidinma Abuwa, Angelina Abbaticchio, Madeline Theodorlis, Deborah Marshall, Crystal MacKay, Cornelia M. Borkhoff, Glen Stewart Hazlewood, Marisa Battistella, Aisha Lofters, Vandana Ahluwalia, Anna R. Gagliardi

**Affiliations:** 1 Toronto General Hospital Research Institute, University Health Network, 200 Elizabeth Street, Toronto, M5G2C4 Canada; 2 University of Calgary, Calgary, Canada; 3 West Park Healthcare Centre, York, Canada; 4 Institute of Health Policy, Management and Evaluation, Dalla Lana School of Public Health, University of Toronto, Toronto, Canada; 5 Department of Family and Community Medicine, University of Toronto, Toronto, Canada; 6 William Osler Health System, Brampton, Canada

**Keywords:** Osteoarthritis, Patient-centred care, Healthcare equity, Women’s health, Disadvantaged groups, Clinical guidelines, Content analysis

## Abstract

**Introduction:**

Women are disproportionately impacted by osteoarthritis (OA) but less likely than men to access early diagnosis and management, or experience OA care tailored through person-centred approaches to their needs and preferences, particularly racialized women. One way to support clinicians in optimizing OA care is through clinical guidelines. We aimed to examine the content of OA guidelines for guidance on providing equitable, person-centred care to disadvantaged groups including women.

**Methods:**

We searched indexed databases and websites for English-language OA-relevant guidelines published in 2000 or later by non-profit organizations. We used manifest content analysis to extract data, and summary statistics and text to describe guideline characteristics, person-centred care (PCC) using a six-domain PCC framework, OA prevalence or barriers by intersectional factors, and strategies to improve equitable access to OA care.

**Results:**

We included 36 OA guidelines published from 2003 to 2021 in 8 regions or countries. Few (39%) development panels included patients. While most (81%) guidelines included at least one PCC domain, guidance was often brief or vague, few addressed exchange information, respond to emotions and manage uncertainty, and none referred to fostering a healing relationship. Few (39%) guidelines acknowledged or described greater prevalence of OA among particular groups; only 3 (8%) noted that socioeconomic status was a barrier to OA care, and only 2 (6%) offered guidance to clinicians on how to improve equitable access to OA care: assess acceptability, availability, accessibility, and affordability of self-management interventions; and employ risk assessment tools to identify patients without means to cope well at home after surgery.

**Conclusions:**

This study revealed that OA guidelines do not support clinicians in caring for diverse persons with OA who face disadvantages due to intersectional factors that influence access to and quality of care. Developers could strengthen OA guidelines by incorporating guidance for PCC and for equity that could be drawn from existing frameworks and tools, and by including diverse persons with OA on guideline development panels. Future research is needed to identify multi-level (patient, clinician, system) strategies that could be implemented via guidelines or in other ways to improve equitable, person-centred OA care.

**Patient or public contribution:**

This study was informed by a team of researchers, collaborators, and thirteen diverse women with lived experience, who contributed to planning, and data collection, analysis and interpretation by reviewing study materials and providing verbal (during meetings) and written (via email) feedback.

**Supplementary Information:**

The online version contains supplementary material available at 10.1186/s12891-023-06877-x.

## Introduction

Women are more likely than men to develop hand, knee and hip osteoarthritis (OA) and experience greater OA severity, pain and functional impairment, all of which exert a considerable psychosocial burden [1–3]. However, women are less likely than men to access the OA care and advice they need, particularly racialized or immigrant women [4–8]. For example,

among 48,218 Canadians, underuse of hip or knee arthroplasty was three times greater in women compared with men despite equal willingness to undergo the procedure [4]. Among 102,767 American women for whom total knee arthroplasty was indicated, Black and Hispanic women were significantly less likely to undergo the procedure even after adjusting for factors including but not limited to age, joint pain, mobility disability, body mass index, number of comorbidities, income education, neighborhood socioeconomic status and geographic region [5].

First-line conservative OA therapy recommendations vary across guidelines but include physical activity, weight management, education for self-management, and pharmacologic and non-pharmacologic pain control when necessary [9, 10]. Second-line therapy may include joint replacement when necessary [9, 10]. Research involving women has identified multiple barriers that influence access to or compliance with recommended management such as pain and fatigue due to OA and self-efficacy to manage OA, both of which limit physical activity [11], and lack of time and prioritization of self-care due to work and family responsibilities [12]. Clinician factors influencing women’s OA care include lack of education in women’s health [13] or OA [14, 15], and gender bias in pain assessment and management [15].

Despite evidence of the disproportionate impact of OA on women, and gendered barriers of access to and quality of OA care, there is little research on strategies (i.e. programs, services, interventions) to overcome those gendered barriers. In 2011, Borkhoff et al. published a review of strategies that support equitable OA care for any disadvantaged group, and only one of 10 eligible studies published up to and including 2009 compared women and men [16]. Nine studies assessed strategies targeting disadvantaged patients, largely via educational self-management programs, but no studies evaluated strategies targeting clinicians. This is an important gap because research shows that educational self-management programs on their own have inconsistent, limited or short-term impact on patient knowledge, self-efficacy, behaviour and OA symptoms [17]. Instead, self-management is more effective when nurtured by physicians who practice person-centred care (PCC) through approaches such as one-on-one patient-provider consultation, tailoring a multi-pronged action plan to patient needs, providing or referring to sources of self-help; and ongoing monitoring and follow-up to assess OA status and goals, and modify the action plan as required [18]. However, clinicians have reported difficulty in providing person-centred OA care and self-management advice [19]. Therefore, solely placing responsibility on patients to self-manage OA is not likely to reduce gendered inequities in OA care, and instead, research is needed on how to support clinicians in providing equitable, person-centred OA care.

Given that clinicians have limited time for continuing professional development [20], and educational meetings have a small impact on clinician knowledge and behaviour [21], an alternative way to support clinicians in providing equitable, person-centred OA care is through clinical guidelines. Clinical guidelines are defined as systematically developed statements to assist practitioner and patient decisions about appropriate health care for specific clinical circumstances [22]. Guidelines play a fundamental role in shaping and improving healthcare delivery and associated patient outcomes, particularly when they include point-of-care tools that help clinicians to implement guideline recommendations in practice [23]. No prior research examined OA guidelines for content that could promote and enable equitable, person-centred OA care. The overall aim of this study was to examine the content of guidelines on OA-relevant topics for recommendations, considerations or other support of equitable, person-centred care for disadvantaged groups including diverse women. If present in at least some guidelines, those could serve as exemplars for other OA guideline developers. If absent, that knowledge represents an opportunity for developers to strengthen their guidelines.

## Methods

### Approach

Content analysis of documents is commonly used to assess the information conveyed in visual or written communication. Specifically, we used manifest content analysis to examine whether and how guidelines on OA topics address equitable, person-centred care [24, 25]. This refers to capturing explicit details without theoretical interpretation. The approach involves organizing content into categories, and then counting, describing and/or comparing categories across documents. While not a typical review, we did search for guidelines, so we complied with the Preferred Reporting Items for Systematic Reviews and Meta-Analyses (PRISMA) [26]. To enhance rigour, multiple team members (CA and AA graduate students, MT research associate, ARG principal investigator) independently analyzed data, and compared data and resolved discrepancies through discussion. Furthermore, a diverse research team reviewed and interpreted data including a 13-member advisory group of diverse women with OA plus healthcare professionals (family physician, rheumatologists, physiotherapist, pharmacist) and health services researchers with expertise in the topics of OA, person-centred care, equity and women’s health. We did not require research ethics board approval because documents were publicly available.

### Eligibility criteria

Additional File 1 describes eligibility criteria in detail. In brief, we included new, updated or adapted/adopted evidence-based English-language guidelines developed by non-profit organizations such as academic groups, governments or governmental agencies, professional societies or charitable foundations on the overall (i.e. across the trajectory of illness) or specific (e.g. pain management) first- or second-line management of all forms of arthritis including OA or OA specifically for adults aged 18+. Evidence-based referred to guidelines informed by a systematic review and critical appraisal of published research. We included guidelines published in 2000 or later, following publication of a National Institutes of Health conference report offering updates on OA care [27, 28]. While guidelines published prior to 2010 may not have been based on up-to-date evidence, including them resulted in a larger sample of guidelines within which to identify possible trends in addressing inequities. We excluded guidelines that did not focus on or include OA, focused solely on alternative medicine therapies, or were based solely on consensus with no review of published research.

### Searching and screening

Additional File 2 shows the strategies used by MT in April 2022 to search for guidelines in indexed databases. We searched MEDLINE and EMBASE using a combination of Medical Subject Headings and keywords. We also searched and browsed the Guidelines International Network Library with the keywords arthritis or osteoarthritis. All results were exported to Excel. To pilot test screening, MT, CA, AA and ARG independently screened 125 titles and abstracts, then compared and discussed screening results to build a common understanding of which titles to include. Thereafter, CA screened remaining titles, and consulted with MT and ARG to resolve uncertainties. CA acquired full-text guidelines, and for each, checked the developer web site to gather complete versions of guidelines or related adjunct documents. CA screened full-text guidelines, consulting with MT and ARG as needed.

### Data extraction

We extracted data on guideline characteristics, and mention of strategies to support person-centred OA care and or equitable access to OA care identified anywhere in the guideline or in documents accompanying the guideline. Characteristics included year of publication, type of developer (academic group, non-profit agency, government, professional society), objective, target audience and whether the development panel included multiple specialties and patients. Person-centred care (PCC) referred to content related to the components of an existing PCC framework that we chose because it was rigorously developed [29], more elaborate than other general PCC frameworks [30], inclusive of approaches deemed essential to person-centred OA care [30–33] and validated through our prior work on what constitutes PCC for diverse women [34, 35]. The framework includes six domains: foster a healing relationship, exchange information, respond to emotions, manage uncertainty, share decisions and enable self-management [29]. Although our primary interest was equitable, person-centred OA care for diverse women, we extracted data pertaining to any disadvantaged group. Equitable access referred to any mention of prevalence of OA among, or challenges or barriers faced in accessing OA care by intersectional factors (e.g. gender, age, ethno-cultural group, socio-economic status or other disadvantaged group), or strategies at any level (e.g. patient, clinician, system) needed or recommended to improve access to and quality of OA care for any disadvantaged group including women. As a pilot test, CA, AA, MT and ARG independently extracted data from three guidelines, then compared and discussed results to establish a shared understanding of data extraction. Thereafter, CA and AA extracted data from remaining guidelines, periodically consulting with MT and ARG to resolve uncertainties, and MT and ARG reviewed all data.

### Data analysis

We used summary statistics to report guideline characteristics, and number of guidelines that discussed PCC domains, intersectional factors, OA prevalence or barriers by intersectional factors and strategies to improve equitable access to OA care overall, and for guidelines published in the most recent decade compared with those published prior. We used summary tables, text and examples extracted from guidelines to describe content related to PCC, OA prevalence or barriers by intersectional factors and strategies to achieve equitable access to OA care.

## Results

### Search results

**Fig. 1:**
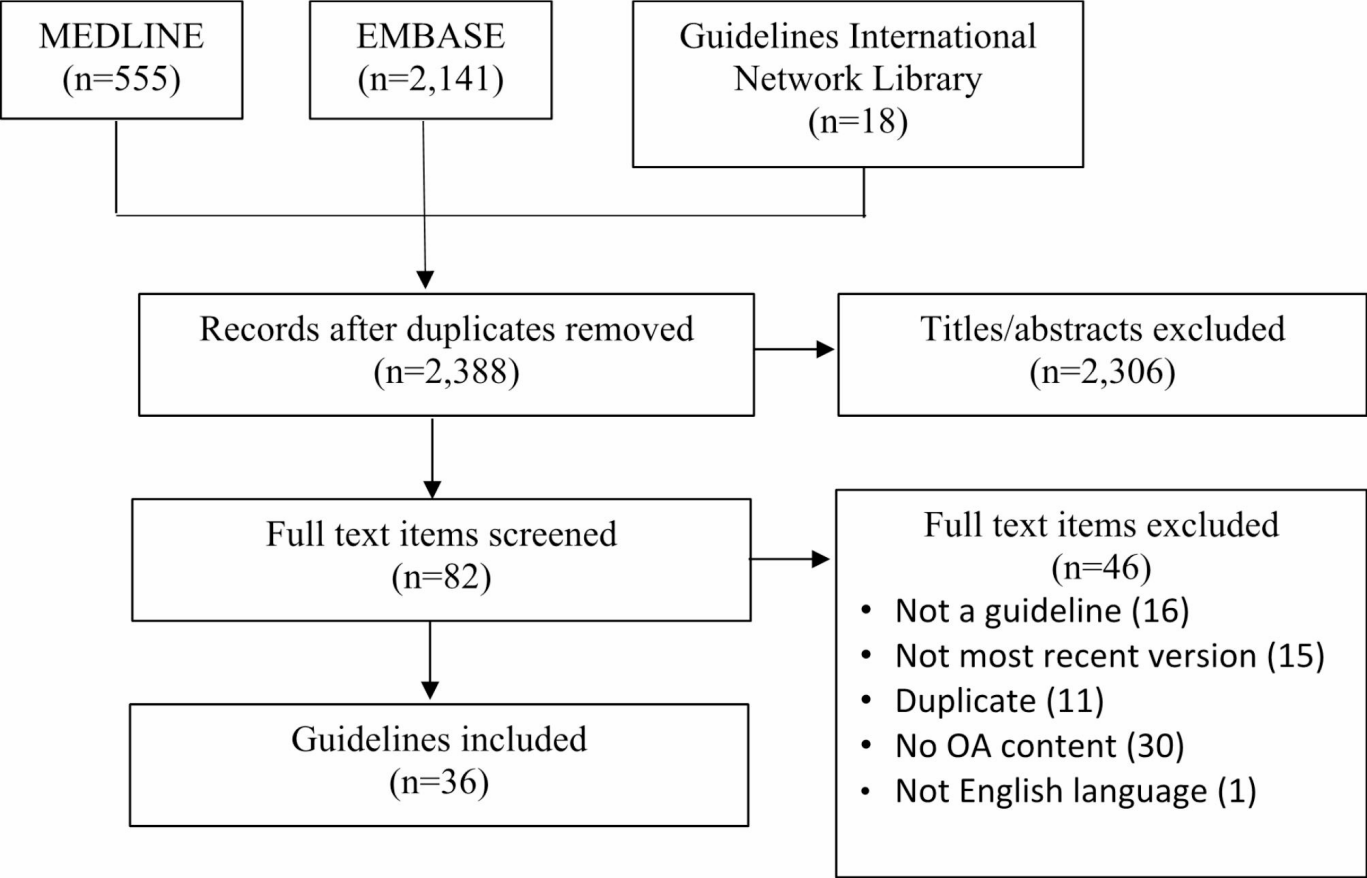
PRISMA diagram. A schematic of the number of search results, screening steps and ultimate number of guidelines included in the study

Figure 1 shows the PRISMA diagram. Searching resulted in 2,696 guidelines, of which 2,388 were unique, and 2,306 were excluded following title and abstract screening. Among 82 full-text guidelines, 46 were excluded because they were not guidelines (16), a more recent or updated version of the guideline was available (15), it duplicated a guideline already included (11), did not address OA (3) or it was not published in English language (1). A total of 36 guidelines were included [9, 10, 35–69].

### Guidelines characteristics

Additional File 3 includes all data on guideline characteristics and Table 1 provides a summary. Guidelines were published between 2003 and 2021, ranged in length from 4 to 669 pages. Guidelines were developed by professional societies (28, 77.7%) or academic groups (8, 22.2%). Most were developed by a consortium of European countries (9, 25.0%), or in the United States (8, 22.2%), Canada (7, 19.4%) or France (6, 16.7%), with 1 (2.8%) each from Turkey, Italy, China, Venezuela and the Netherlands. Guidelines addressed the following types of OA: a combination of hand, hip or knee (12, 33.3%), knee (10, 27.7%), all types (8, 22.2%,) hip (4, 11.1%), hand (1, 2.8%) or shoulder (1, 2.8%). Most focused on overall management of OA (17, 47.2%), while others focused on specific topics, including physical activity or exercise (10, 27.8%), physical therapy (2, 5.6%), diagnosis (2, 5.6%), pain management (1, 2.8%), patient education (1, 2.8%), and use of braces (1, 2.8%), taping (1, 2.8%) or foot orthotics (1, 2.8%). Many guidelines were targeted at healthcare professionals like rheumatologists or physiotherapists (16, 44.4%), and some were created for use by healthcare professionals and patients (10, 27.8%). Others (10, 27.8%) did not report target audience. Most (34, 94.4%) guidelines were based on systematic review and stakeholder consultation, and 2 (5.6%) on systematic review only. All guidelines were developed by multidisciplinary panels; few (14, 38.9%) development panels included patients. Most developers employed custom means to assess evidence (21, 58.3%); others used GRADE (5, 13.9%), AGREE (4, 11.1%), or the Oxford (4, 11.5%) or Ottawa Panel (2, 5.6%) system for grading evidence.

**Table 1:** Summary of characteristics of included guidelines

Guideline [reference]	Year published	Country/ Region	Number of pages	Developer type	Objective (OA site, scope)	Methods	Development panel
American Academy of Orthopedic Surgeons [9]	2021	United States	126	Professional society	Knee, overall non-surgical	Systematic review and stakeholder consultation	Multiple specialties
American College of Rheumatology/Arthritis Foundation [10]	2020	United States	19	Professional society	Hand, hip, knee; overall	Systematic review and stakeholder consultation	Multiple specialties and patients
American Physical Therapy Association [36]	2020	United States	29	Professional society	Knee, physical therapy	Systematic review and stakeholder consultation	Multiple specialties and patients
American Academy of Orthopaedic Surgeons [37]	2020	United States	76	Professional society	Shoulder, overall	Systematic review and stakeholder consultation	Multiple specialties
Rheumatology and Immunology Expert Committee of the Cross-Strait Medical and Health Exchange Association [38]	2020	China	19	Professional society	All, overall	Systematic review and stakeholder consultation	Multiple specialties
American Academy of Orthopaedic Surgeons [39]	2020	United States	4	Professional society	Hip, overall	Systematic review and stakeholder consultation	Multiple specialties
The Italian Society for Rheumatology [40]	2019	Italy	17	Professional society	Hand, hip, knee; overall	Systematic review and stakeholder consultation	Multiple specialties
Osteoarthritis Research Society International [41]	2019	United States	12	Academic group	Knee, hip; overall non-surgical	Systematic review and stakeholder consultation	Multiple specialties
European Society for Clinical and Economic Aspects of Osteoporosis, Osteoarthritis and Musculoskeletal Diseases (ESCEO) [42]	2019	Europe	14	Professional society	Knee, overall	Systematic review and stakeholder consultation	Multiple specialties and patients
European Alliance of Associations for Rheumatology (EULAR) [43]	2019	Europe	9	Professional society	Hand, overall	Systematic review and stakeholder consultation	Multiple specialties and patients
European Alliance of Associations for Rheumatology (EULAR) [44]	2018	Europe	11	Professional society	All, pain management	Systematic review and stakeholder consultation	Multiple specialties and patients
European Alliance of Associations for Rheumatology (EULAR) [45]	2018	Europe	10	Professional society	All, physical activity	Systematic review and stakeholder consultation	Multiple specialties and patients
The Ottawa Panel [46]	2017	Canada	14	Academic group	Knee, physical activity	Systematic review and stakeholder consultation	Multiple specialties and patients
The Ottawa Panel [47]	2017	Canada	16	Academic group	Knee, physical activity	Systematic review and stakeholder consultation	Multiple specialties and patients
The Ottawa Panel [48]	2017	Canada	13	Academic group	Knee, physical activity	Systematic review and stakeholder consultation	Multiple specialties and patients
American Physical Therapy Association [49]	2017	United States	37	Professional society	Hip, physical activity	Systematic review and stakeholder consultation	Multiple specialties and patients
European Alliance of Associations for Rheumatology (EULAR) [50]	2017	Europe	11	Professional society	All, diagnosis	Systematic review and stakeholder consultation	Multiple specialties and patients
Turkish League Against Rheumatism (TLAR) [51]	2017	Turkey	17	Professional society	All, overall	Systematic review and stakeholder consultation	Multiple specialties
Pan-American League of Associations for Rheumatology (PANLAR) [52]	2016	Venezuela	10	Professional society	Hand, hip, knee; overall	Systematic review and stakeholder consultation	Multiple specialties and patients
The Ottawa Panel [53]	2016	Canada	12	Academic group	Hip, physical activity	Systematic review and stakeholder consultation	Multiple specialties
American Academy of Orthopaedic Surgeons [54]	2015	United States	669	Professional society	Knee, overall surgical	Systematic review and stakeholder consultation	Multiple specialties
European Alliance of Associations for Rheumatology (EULAR) [55]	2013	Europe	11	Professional society	Knee, hip; overall non-surgical	Systematic review and stakeholder consultation	Multiple specialties and patients
The Ottawa Panel [56]	2012	Canada	17	Academic group	All, physical activity	Systematic review	Multiple specialties
Pan-American League of Associations for Rheumatology (PANLAR) [57]	2011	The Netherlands	15	Professional society	Knee, hip; physical therapy	Systematic review and stakeholder consultation	Multiple specialties
The Ottawa Panel [58]	2011	Canada	19	Academic group	All, overall	Systematic review	Multiple specialties
The Ottawa Panel [59]	2011	Canada	41	Academic group	All, education	Systematic review and stakeholder consultation	Multiple specialties and patients
European Alliance of Associations for Rheumatology (EULAR) [60]	2010	Europe	7	Professional society	Knee, diagnosis	Systematic review and stakeholder consultation	Multiple specialties
The French Physical Medicine and Rehabilitation Society (SOFMER) [61]	2009	France	8	Professional society	Knee, bracing	Systematic review and stakeholder consultation	Multiple specialties
The French Physical Medicine and Rehabilitation Society (SOFMER) [62]	2008	France	4	Professional society	Knee, hip; taping	Systematic review and stakeholder consultation	Multiple specialties
The French Physical Medicine and Rehabilitation Society (SOFMER) [63]	2008	France	7	Professional society	Knee, hip; foot orthotics	Systematic review and stakeholder consultation	Multiple specialties
The French Physical Medicine and Rehabilitation Society (SOFMER) [64]	2007	France	6	Professional society	Knee, hip; physical activity	Systematic review and stakeholder consultation	Multiple specialties
The French Physical Medicine and Rehabilitation Society (SOFMER) [65]	2007	France	10	Professional society	Knee, hip; physical activity	Systematic review and stakeholder consultation	Multiple specialties
The French Physical Medicine and Rehabilitation Society (SOFMER) [66]	2007	France	9	Professional society	Knee, hip; overall surgical	Systematic review and stakeholder consultation	Multiple specialties
British Society for Rheumatology [67]	2005	United Kingdom	7	Professional society	Knee, hip; physical activity	Systematic review and stakeholder consultation	Multiple specialties
European Alliance of Associations for Rheumatology (EULAR) [68]	2005	Europe	15	Professional society	Hip, overall	Systematic review and stakeholder consultation	Multiple specialties
European Alliance of Associations for Rheumatology (EULAR) [69]	2003	Europe	11	Professional society	Knee, overall	Systematic review and stakeholder consultation	Multiple specialties

### Person-centred OA care

Additional File 4 includes all data on PCC and Table 2 provides a summary. Most (29, 80.5%) guidelines included content relating to at least one PCC domain. Seven (19.4%) included no PCC content [13,17,20,22–24,31,33], and no single guideline addressed all 6 PCC domains. This did not notably change over time: included guidelines that were published in the most recent decade (2014 to 2021) addressed a median of 2 PCC domains and those published prior to 2014 (2003 to 2013) addressed a median of 1 PCC domain. The most frequently mentioned domains were “enable self-management” (27, 75.0%) and “share decisions” (21, 58.3%). Few guidelines addressed the domains “exchange information” (10, 27.7%), fewer still included content relevant to “respond to emotions” (3, 8.3%) and “manage uncertainty” (1, 2.8%), and none considered “foster a healing relationship”.

**Table 2:** Summary of person-centred care content in included guidelines

PCC Domain [29, 34, 35]	Guidelines (n, %) [references]	Examples [reference, page number]	
		Limited content	Expanded content
**Foster a healing relationship** Establishing a friendly, courteous, and comfortable relationship	(0, 0)	--	--
**Exchange information** Learning about the patient; words or language used to discuss health care	(9, 25.0) [9, 40, 41, 44, 45, 49, 54, 55, 57]	The treatments and procedures for each patient relies on mutual communication between the patient, physician, and other healthcare professionals [54 p4]	Invite patients disclosing the impact of pain on their daily functioning, to assess their ideas and concerns regarding the cause of their pain and the perceived control over pain episodes, and to take account of their expectations and preferences for treatment. It is deemed important to establish the patient’s functional and valued life goals, that is, what it is that they cannot currently do as well as they would wish to. Assess sleep problems: the quantity and quality of sleep, including whether the patient feels refreshed on waking and sleep hygiene habits such as regular exercise during the day, stress management, noise, sleep timing and avoidance of caffeine, nicotine, alcohol and daytime napping. Assess social factors related to pain and its consequences: the way family members and other significant others react to patient’s pain or pain-related disability; work; family and friends; economic problems; housing. Assess other factors that might influence pain or pain management, such as dependence on tobacco, alcohol or drugs [44 p802]
**Respond to emotions** Responding to or managing emotional reactions	(3, 8.3) [9, 44, 57]	Preoperative education could be considered if there is much anxiety for the operation [57 p274]	Assess beliefs and emotions about pain and pain-related disability: the psychological response to pain and psychological vulnerability factors, psychological distress, psychiatric comorbidity and cognitions such as catastrophizing (rumination, magnification and helplessness), fear of movement-related pain, catastrophizing and pain self-efficacy. If there are indications that social variables or psychological factors interfere with effective pain management and functional status, then consider (depending on the severity) providing basic social and psychological management support or referral to a psychologist, social worker, self-management support programme, CBT or multidisciplinary treatment. If psychopathology (e.g., depression and anxiety) is present, discuss treatment options with the patient and the patient ’s primary care physician. If psychosocial factors such as fear of movement or catastrophising cognitions underlie a disabled, sedentary lifestyle, then consider a multidisciplinary intervention including cognitive-behavioural therapy [44 p802-803]
**Manage uncertainty** Addressing uncertainties about prognosis or outcomes	(1, 2.7) [41]	Clinicians are encouraged to continually provide their patients with necessary information about OA disease progression and self-care techniques and to promote hope, optimism, and a positive expectation of benefit from treatment [41 p1583]	--
**Share decisions** Engaging patient in discussion and decision-making. Including planning care with patients and tailoring plan to diverse characteristics, including patient characteristics, preferences, circumstances (e.g., finances)	(22, 61.1) [9, 10, 36, 37, 39–45, 47, 49–55, 65–69]	Treatment decisions should be made in light of all circumstances presented by the patient [54 p4]	Each health professional must decide with each patient the most appropriate management plan at a particular time and for that location. Many patient-centred factors are important in determining the selection of treatments for individual patients with knee OA—for example, psychosocial factors and OA status; comorbid disease and drugs; patient beliefs about their knee OA; patient beliefs and preferences for its management; and previous patient experiences of treatments and health professionals. The management plan for patients with knee OA has to be individualised, reviewed, and adjusted in the light of the patient’s response and adherence and will vary between patients and between locations [69 p1154]
**Enable self-management** Setting expectations for follow-up care; preparing for self-managing health and well-being	(27, 75.0) [9, 10, 36, 38–59, 64–69]	Self-management programs are recommended to improve pain and function for patients with knee osteoarthritis [9 p8]	Teach and encourage behavioural change strategies through goal setting of physical activity and weight changes, action plans to maintain changes and regular follow-up over at least 1 year to re-evaluate and discuss goals and action plans…the addition of advice from a dietician for overweight or obese patients to the combination of patient education or self-management intervention plus exercise was found to improve both pain and function in patients with hip or knee OA [55 p1128]

Even when mentioned, guidance in or accompanying the guideline on how clinicians could support person-centred OA care was often brief or vague. For example, many guidelines acknowledged the domain of “share decisions”, yet offered little to no details on how this could be achieved: “Treatment decisions should be made in light of all circumstances presented by the patient” [54]. Similarly, for the domain of “respond to emotions”, guidelines offered little instruction on how to assess, respond to or address anxiety, stress or other concerns: “Preoperative education could be considered if there is much anxiety for the operation” [57].

### Equitable access to OA care

Additional File 5 includes all data on OA prevalence and barriers of OA care by intersectional factors, and Table 3 provides a summary. Among 36 guidelines, 14 (38.8%) acknowledged greater prevalence of OA by intersectional factors: older age (13, 36.1%), gender, referring to women (11, 30.5%), lower socioeconomic status (1, 2.7%), and geographical location (1, 2.7%). No guidelines noted that the burden of OA is greater among racialized or immigrant women. This did not notably change over time: included guidelines that were published in the most recent decade (2014 to 2021) addressed a median of 1 equity domains and those published prior to 2014 (2003 to 2013) addressed a median of 0 equity domains. Even when guidelines mentioned the disproportionate burden of OA on diverse persons, details were limited; for example: “prevalence [for hip and knee OA] was higher for females than males” [49]. Only 3 (8.3%) guidelines acknowledged that lower socioeconomic status contributed to barriers or challenges in accessing OA care. For example, one guideline briefly mentioned that self-management programs may not be accessible to patients: “Self-management programs are feasible for patients provided they have appropriate access. Some patients may have limited access for participation, making the programs less feasible” [9].

**Table 3:** Summary of content on OA prevalence and barriers by intersectional factors

Factors	Guidelines (n, %) [references]	Examples [reference, page number]	
		Limited	Expanded
**Prevalence by intersectional factors**			
Age	(13, 36.1) [9, 35–38, 42, 43, 49, 52, 55, 56, 59, 60, 69]	Hand osteoarthritis (OA) is a common musculoskeletal disease, with prevalence rising steeply with increasing age [43 p16]	The incidence of OA increased significantly with age (4), 10–17% in the population over 40 years old, 50% in the population over 60 years old, and 80% in the population over 75 years old, and the disability rate was 53% [38 p2]
Gender	(11, 30.5) [9, 35–38, 49, 52, 53, 56, 59, 60, 69]	Prevalence [for hip and knee OA] was higher for females than males [49 p7]	Risk factors of [osteoarthritis] increase with age, especially in women. Although women represent 51% of the general population in the United States, they represent 78% of the patients diagnosed with osteoarthritis between 2008 and 2014 [9 p16]
Socioeconomic status	(1, 2.7) [49]	Living in a community with a high poverty level is independently associated with radiographic OA in 1 or both hips. Low education attainment is independently associated with symptomatic OA of 1 or both hips…age, history of hip developmental disorders, previous hip joint injury, reduced hip ROM (especially hip IR), presence of osteophytes, lower socioeconomic status, higher bone mass, and higher BMI are risk factors for developing hip OA [49 p8]	--
Geography	(1, 2.7) [38]	The incidence of OA is… higher in rural areas than in urban areas [38 p2]	--
**Barriers or challenges by intersectional factors**			
Socioeconomic status	(3, 8.3) [9, 41, 47]	Self-management programs are feasible for patients provided they have appropriate access. Some patients may have limited access for participation, making the programs less feasible [9 p35]	--

### Strategies to improve equitable access to OA care

Additional File 5 includes all data on strategies to improve equitable access to OA care, and Table 4 provides a summary. Only 2 (5.5%) guidelines recommended one strategy each to enhance access to OA care for disadvantaged groups. One guideline included a patient-level strategy related to self-management, recommending that clinicians explore with patients the acceptability, availability, accessibility, and affordability of self-management interventions, noting that many communities offer free programs [10]. Another guideline offered a clinician-level strategy related to pre-operative assessment of rehabilitation requirements, recommending that clinicians use existing risk assessment tools to identify disabilities or precarious social conditions, and anticipate difficulties in returning home following arthroplasty to allow for better preparation [66]. No guidelines mentioned system-level strategies to improve equitable access to OA care.

**Table 4:** Summary of content on strategies to improve equitable access to OA care

Strategy level	Strategy type	Guidelines (n,%) [references]	Examples
Patient Offered to persons with OA to improve knowledge, confidence, behaviour, OA symptoms, OA status, or quality of life	Self-management advice	(1, 2.7) [10]	Exercise recommendations to patients should focus on the patient’s preferences and access, both of which may be important barriers to participation. If a patient does not find a certain form of exercise acceptable or cannot afford to participate or arrange transportation to participate, he or she is not likely to get any benefit from the suggestion to pursue that exercise…the availability, accessibility, and affordability of some [educational, physical, behavioral, psychosocial, mind-body, and pharmacologic] interventions vary, but in many communities the Arthritis Foundation, as well as local hospitals and other health-related agencies, offer free self-efficacy and self-management programs [p154,160]
Clinician Offered to healthcare professionals to improve knowledge, confidence, behaviour, or how they provide OA care (e.g., skills)	Clinical assessment tools	(1, 2.7) [66]	Rehabilitation requires a preoperative evaluation of patient needs and referral to a qualified health care professional in the patient’s education and preparation for the return home… Recommendation to perform preoperative needs analysis of patients [is suggested, especially for] a more fragile population in terms of difficulties in returning home, presurgical major disability, or precarious social conditions or comorbidities…the use of a predictive orientation questionnaire such as the Risk Assessment and Predictor Tool could allow for better preparation [66 p196]
System Developed and/or offered by health systems or government to improve access to OA care, advice and support	--	--	--

## Discussion

This study examined the content of 36 clinical guidelines relevant to OA published between 2003 and 2021, revealing limited guidance for clinicians on how to achieve equitable, person-centred care for women with OA, or other disadvantaged groups. While most guidelines mentioned concepts related to “enable self-management” (75.0%) and “share decisions” (58.3%), few addressed “exchange information”, “respond to emotions” or “manage uncertainty”, and none offered guidance on “foster a healing relationship”. Perhaps more importantly, even when mentioned, details were limited and vague, providing clinicians with no concrete actions to implement. Few (38.9%) guidelines noted greater prevalence of OA or barriers to accessing OA care among any disadvantaged group (3 specifically stating that socioeconomic status was a barrier), and only 2 (5.6%) offered guidance to clinicians on how to improve equitable access to OA care. Guideline content reflecting PCC and equity did not increase over time.

These findings are consistent in general with considerable prior research that evaluated the quality of clinical guidelines and identified limitations in content [70, 71], particularly with respect to guidance for clinicians on how to implement the recommendations [72]. These findings are also similar to our prior research showing that guidelines on health issues with known gendered inequities (e.g. depression, cardiac rehabilitation) failed to offer practical guidance on how to achieve PCC for women [73]. In OA-specific research, analysis of 20 OA guidelines identified 11 as high quality, from which authors extracted common recommendations, with one being that care should be patient-centred [74]. Another review of 17 physical therapy guidelines for OA identified variations in interventions, levels of evidence, and strength of recommendations across the guidelines [75]. Another appraisal of 17 guidelines on the non-pharmacological management of OA found that few guidelines addressed all quality domains, and the overall quality according to a 7-point scoring system was 4.8 ± 0.41 [76]. While these OA-specific studies analyzed guideline content, our findings are novel because no prior research specifically analyzed OA guideline content for advice to clinicians on how to achieve equitable, person-centred OA care for women, or for other disadvantaged persons.

These findings raise several implications for guideline development. The first is to strengthen guidelines with guidance for person-centred OA care. Most guidelines included in this review did not address “exchange information”, “respond to emotions” or “manage uncertainty”, and none offered guidance to “foster a healing relationship.” Insight on how to do so could be gained from general frameworks and syntheses on approaches for PCC [29, 30], our prior research on what constitutes PCC for diverse women [34, 35], and OA-specific PCC frameworks and standards, and patient-reported quality indicators for OA [30–33]. For example, based on the views of diverse women, guidance for establishing a healing relationship might include: who interest in the person through friendly conversation prior to discussion of clinical issues; assume a non-judgmental attitude by maintaining a neutral disposition and speaking in a respectful manner; and display attentiveness by making eye contact and avoiding prolonged computer use [34, 35]. It may also be important to assess the content of OA guidelines published subsequent to this review for content relevant to inequities. For example, the October 2022 update of the National Institute for Health and Care Excellence OA guideline noted that: OA information and support must be tailored to individual needs including language and culture; and those who vary by age, sex or gender, or other factors should not be excluded from referral for joint replacement consultation [77]. A complementary approach to making guidelines more patient-oriented is to include and meaningfully diverse women or persons representing other disadvantaged groups in panels that develop clinical guidelines and/or identify patient preferences in published research. Notably, this study found that few (14, 38.9%) developers engaged patients in guideline development; however, doing so is widely recommended [78], guidance is available to support patient engagement in guideline development [78–81], and there is evidence that engaging patients in guideline development leads to greater use and impact of guidelines [82, 83].

Another way to strengthen OA guidelines is to include content that raises clinician awareness of the barriers faced by women, or other disadvantaged groups, in accessing OA care, and offers concrete guidance to clinicians on how to facilitate equitable access to OA care. In this study, only 2 (5.6%) included such guidance: one guideline recommended that clinicians assess the acceptability, availability, accessibility and affordability of self-management programs and refer patients to free programs that are available in many communities [10] and another guideline recommended that clinicians use assessment tools to identify patients requiring functional and social support at home following surgery [66]. Primary research is needed to identify additional multi-level (patient, clinician, system) strategies that could be implemented to enhance access to OA care, and to add patient- and clinician-level strategies to OA guidelines, likely through interviews with key informants including women with OA, clinicians, and healthcare managers and policy-makers. In the meantime, clinicians could refer to existing PCC guidance to improve discussions with patients about self-management [28–33], and use the Risk Assessment and Prediction Tool (RAPT) after hip or knee arthroplasty [84]. Research is needed to assemble other such tools that help clinicians to identify women or other disadvantaged persons with OA who require tailored assistance. A final way to enhance equity issues in OA guidelines is to strengthen the evidence upon which guidelines are based by recruiting women and other disadvantaged persons in randomized controlled trials, which is a complex issue beyond the scope of this research that may take time and concerted effort to address. Apart from enhancing OA guidelines, clinicians would benefit from medical education and continuing professional development on person-centred care for women as our prior content analysis of curriculum at 16 Canadian medical schools identified little exposure to the concepts of person-centred care or womens health [85, 86]. Given likely variations in approaches to person-centred care or womens health worldwide, strategies other than guidelines, education and continuing professional development may be required to improve access to and quality of OA care for diverse persons.

Strengths of this study include the use of rigorous methods for searching, screening and content analysis [24, 25], and complying with standards for conducting reviews [26]. We organized and interpreted data according to an established six-domain PCC framework that encompassed OA-specific PCC recommendations [29–33]. We included guidelines developed internationally, increasing the broad relevance of the findings. To further enhance relevance, even though our interest is gendered disparities, we extracted content related to any disadvantaged group. Regarding reliability, multiple researchers independently undertook specific tasks (i.e. screening, data extraction), we incorporated feedback throughout the study from our inter-disciplinary research team that included a 13-member advisory group of diverse women with OA, and the research team reviewed the findings. Some limitations must be mentioned. As with any review, the search strategy we employed may have failed to identify all relevant guidelines, and the search was restricted to English-language guidelines. We did not assess methodological quality of included guidelines as we focused on content that might reduce disparities in OA care rather than general guideline quality. Findings may have differed had we used a PCC framework other than the one employed [29–33].

## Conclusions

We examined the content of 36 OA guidelines for advice to clinicians on how to achieve equitable, person-centred OA care for women and those of other groups facing disparities in OA care. Few guidelines comprehensively addressed PCC or equity issues, meaning that current guidelines do not support clinicians in caring for diverse persons with OA who face disadvantages due to intersectional factors that influence access to and quality of care. Given the prevalence and profound impact of OA, and evidence that women are not getting recommended care, action is urgently needed to enhance guidelines as one approach for helping clinicians to optimize OA care, and by doing so, improve wellness and quality of life for diverse persons with OA. While the results are disappointing, they do reveal ways to strengthen OA guidelines by incorporating guidance for PCC and for equity that could be drawn from existing frameworks and tools, and by including women with OA and other persons representing disadvantaged groups on guideline development panels. Future research is needed to identify multi-level (patient, clinician, system) strategies that could be implemented via guidelines or in other ways to improve equitable, person-centred OA care.

### Electronic supplementary material

Below is the link to the electronic supplementary material.

**Additional File 1**. OA guideline eligibility criteria

**Additional File 2**. OA guideline search strategies

**Additional File 3**. Characteristics of included guidelines

**Additional File 4**. Data extracted from included guidelines on person-centred care

**Additional File 5**. Data extracted from included guidelines on strategies to achieve equitable access to OA care

## Data Availability

All data generated or analysed during this study are included in this published article and its supplementary information files.

## Abbreviations

OA: Osteoarthritis
PCC: Person-centred care
