# Supplementary material for: Identifying strategies that support equitable person-centred osteoarthritis care for diverse women: content analysis of guidelines
Source: BMC Musculoskelet Disord. 2023 Sep 14;24:734. doi: 10.1186/s12891-023-06877-x (PMC10500823; doi:10.1186/s12891-023-06877-x)
Supplement: Supplementary file 1 — Additional File 1. OA guideline eligibility criteria [file 12891_2023_6877_MOESM1_ESM.docx]

**Additional File 1. OA guideline eligibility criteria**

**Definition**

“Guidelines” refers to new, updated or adapted/adopted documents developed using standardized methods by non-profit organizations (e.g., academic groups, government, governmental agencies, professional societies, charities/foundations) that include recommendations informed by a systematic review of evidence and an assessment of the benefits and harms of alternative care options. Guidelines are used by healthcare professionals to guide their care of patients, or by healthcare managers or policymakers to inform decisions about organizing and funding healthcare services.

**ELIGIBLE**

**People/Population**

Guidelines offer recommendations for the care of adults aged 18+ with:

- Arthritis (and include sections for different types of arthritis including osteoarthritis) or osteoarthritis arthrosis or arthropathy or degenerative joint disease in general or overall, across the trajectory of illness (e.g., prevention, diagnosis, first line treatment, second line treatment)
- Osteoarthritis affecting specific joints (e.g., guideline may be specific to knee or hip osteoarthritis)
- Specific aspect of care for osteoarthritis (e.g., pain management, joint replacement, post-operative management)
- Treatments include:
  - First line treatment:
    - Education/counseling about OA and/or about how to self-manage OA (e.g., counselling, courses, information)
    - Encourage increased physical activity
    - Encourage healthy diet
    - Encourage weight loss
    - Physiotherapy
    - Occupational therapy
    - Massage therapy
    - Pharmacologic and non-pharmacologic means of managing pain
      - Includes integrative medicine (coordinated use of conventional and complementary medicine; see definitions below)
  - Second line treatment:
    - Injections (e.g., corticosteroids, hyaluronic acid)
    - Arthroscopic surgery (minimally invasive procedure to examine and sometimes correct damage in a joint)
    - Osteotomy (surgical procedure to shorten or lengthen a bone to adjust its alignment)
    - Arthrodesis/joint fusion (fusion of two bones to lessen pain and create more stable joint)
    - Joint replacement (e.g., of any of the body areas noted above)

**Issue/Intervention**

- Issue: guidelines as a type of document or publication that would be used by clinicians to inform how they care for patients with potential or confirmed OA; clinicians include physicians of different specialties, nurses, nurse practitioners, physiotherapists, etc.
- Developed in any country by non-profit organizations (e.g., professional societies, academic institutions, government agencies, disease-specific foundations, or quality improvement/monitoring agencies)
- Developers may also include international groups (e.g., entities that include membership from two or more countries)
- Developed based on systematic review and critical appraisal of published research. May involve consensus and be labelled as a consensus guideline provided a review of published research served as the basis for consensus
- Published in English language
- Published from the year 2000 onwards to capture clinical recommendations that reflect current research

**Comparisons**

- May compare the effectiveness of different care options by describing the underlying evidence and issuing recommendations based on the strength of that evidence

**Outcomes**

- Include any reported by the guideline, but may include patient preferences, quality of life and clinical outcomes associated with different types of care, for example, pain relief, mobility, etc.

**NOT ELIGIBLE**

- Guidelines focused on rheumatoid arthritis
- Guidelines that are not publicly available
- Guidelines based solely on consensus (i.e. expert opinion) with no review of published research
- Guidelines that solely focus on OA prevention or treatment with alternative medicine (may be referred to as oriental or complementary medicine)
  - Alternative medicine – non-mainstream practice used in place of conventional medicine
  - Complementary medicine – non-mainstream practice used together with conventional medicine (e.g., herbal medicines, acupuncture, spinal manipulation, Tai Chi, massage, yoga)
- Guidelines that pertain to laboratory testing
- Publication type: protocols, proceedings, abstracts, letters, editorials, case reports, case studies, dissertation, book chapters or commentaries, reviews or comparisons of guidelines, but we will screen references for eligible guidelines
