# Supplementary material for: Identifying strategies that support equitable person-centred osteoarthritis care for diverse women: content analysis of guidelines
Source: BMC Musculoskelet Disord. 2023 Sep 14;24:734. doi: 10.1186/s12891-023-06877-x (PMC10500823; doi:10.1186/s12891-023-06877-x)
Supplement: Supplementary file 2 — Additional File 2. OA guideline search strategies [file 12891_2023_6877_MOESM2_ESM.docx]

**Additional File 2. OA guideline search strategies**

MEDLINE

| **Searches** | **Results** |  |
| --- | --- | --- |
| 1 | exp Osteoarthritis/ | 71752 |
| 2 | Arthritis/ | 36505 |
| 3 | 1 or 2 | 106480 |
| 4 | guideline/ or practice guideline/ | 36944 |
| 5 | guidelines as topic/ or practice guidelines as topic/ | 168926 |
| 6 | 4 or 5 | 204469 |
| 7 | 3 and 6 | 694 |
| 8 | limit 7 to (english language and humans) | 611 |
| 9 | limit 8 to (comment or editorial or english abstract or letter) | 56 |
| 10 | 8 not 9 | 555 |

EMBASE

Database(s): **Embase Classic+Embase**1947 to 2022 April 08 
Search Strategy:

| **#** | **Searches** | **Results** |
| --- | --- | --- |
| 1 | exp Osteoarthritis/ | 155738 |
| 2 | Arthritis/ | 85428 |
| 3 | 1 or 2 | 233999 |
| 4 | guideline/ or practice guideline/ | 494183 |
| 5 | guidelines as topic/ or practice guidelines as topic/ | 453519 |
| 6 | 4 or 5 | 494227 |
| 7 | 3 and 6 | 3333 |
| 8 | limit 7 to (human and english language) | 2979 |
| 9 | limit 8 to (conference abstract or editorial or letter) | 838 |
| 10 | 8 not 9 | 2141 |
