# Supplementary material for: Identifying strategies that support equitable person-centred osteoarthritis care for diverse women: content analysis of guidelines
Source: BMC Musculoskelet Disord. 2023 Sep 14;24:734. doi: 10.1186/s12891-023-06877-x (PMC10500823; doi:10.1186/s12891-023-06877-x)
Supplement: Supplementary file 3 — Additional File 3. Characteristics of included guidelines [file 12891_2023_6877_MOESM3_ESM.docx]

**Additional File 3. Characteristics of included guidelines**

| Title Organization Year [Reference] Focus | Content (pages and structure, etc.) | Developer type | Objective | Target audience | Development approach |
| --- | --- | --- | --- | --- | --- |
| Management of Osteoarthritis of the Knee (Non-Arthroplasty) (3^rd^ Edition)  American Academy of Orthopedic Surgeons  2021 [9]  Management of OA | 126-page document including: summary of recommendations, introduction, methods, recommendations, Appendix I-IV | Professional society | Provides recommendations that will help practitioners to integrate the current evidence and clinical practice, and it highlights gaps in the literature in need of future research. It also serves as an information resource  for developers and applied users of clinical practice guidelines (p.15) | Orthopaedic surgeons, appropriately trained physicians and clinicians who manage the treatment of OA of the knee (p.15) | This clinical practice guideline was prepared by the AAOS Osteoarthritis of the Knee Guideline physician development group (clinical experts) with the assistance of the AAOS Clinical Quality and Value (CQV) Department (methodologists). This involved the systematic review with a comprehensive search of the literature. The recommendations and their strength were then voted on by the guideline development group members (p.17, p.18) |
| 2019 American College of Rheumatology/Arthritis Foundation Guideline for the Management of Osteoarthritis of the Hand, Hip, and Knee  American College of Rheumatology/Arthritis Foundation  2020 [10]  Management of OA | 14-page document including: introduction, methods, results and recommendations (comprehensive management of OA; physical, psychosocial, and mind-body approaches; and pharmacologic management) and a discussion | Professional society | To update the 2012 American College of Rheumatology recommendations for the management of hand, hip, and knee osteoarthritis (OA) through an evidence-based guideline for the comprehensive management of (OA) of the hand, hip, and knee (p.149) | Clinicians and patients (p.149) | A Literature Review Team performed a systematic literature review to summarize evidence supporting the benefits and harms of available educational, behavioral, psychosocial, physical, mind-body, and pharmacologic therapies for OA. Grading of Recommendations Assessment, Development and Evaluation (GRADE) methodology was used to rate the quality of the evidence. A Voting Panel, including rheumatologists, an internist, physical and occupational therapists, and patients, achieved consensus on the recommendations. This followed the American College of Rheumatology guideline development process (p.150) |
| Physical Therapist Management of Total Knee Arthroplasty  American Physical Therapy Association  2020 [36]  Management of total knee arthroplasty | 29-page document including: overview, methods, recommendations | Professional society | Addresses the management of adult patients with knee osteoarthritis undergoing primary total knee arthroplasty. In addition to providing practice recommendations, this guideline also highlights limitations in the literature, areas that require future research, intentional vagueness, and quality improvement activities (p.1604) | All qualified and appropriately trained physical therapists, decision makers and developers of practice guidelines (p.1604) | This clinical practice guideline was developed by an American Physical Therapy (APTA) volunteer guideline development group (GDG) that consisted of physical therapists, an orthopedic surgeon, a nurse, and a consumer (p.1603). The GDG defined the scope by creating PICO(T) questions (population, intervention, comparison, outcome, and time) that directed the literature search. The GDG performed final reviews of recommendations, provided rationale in the context of physical therapist practice, and adjusted the strength of the recommendations depending on the magnitude of benefit, risk, harm, and cost (p.1605) |
| Management of Glenohumeral Joint Osteoarthritis  American Academy of Orthopaedic Surgeons  2020 [37]  Management of OA | 76-page document including: a summary of recommendations, summary of consensus statements, introduction, methods, and recommendations | Professional society | To provide practice recommendations based on the best available evidence, highlight the limitations in the current literature, and suggest areas for future research (p.14) | Health care professionals and developers of guidelines (p.14) | This clinical practice guideline was prepared by the AAOS Management of Glenohumeral Joint Osteoarthritis Clinical Practice Guideline Physician Development Group (clinical experts) with the assistance of the AAOS Clinical Quality and Value (CQV) Department (methodologists). A systematic review was carried out while defining the scope of the clinical practice guideline by creating PICO Questions (i.e., population, intervention, comparison, and outcome) that directed the literature search. The resulting recommendations and their strength were voted on by the guideline development group Members (p.18) |
| Guidelines for the diagnosis and treatment of osteoarthritis in China (2019 edition)  Rheumatology and Immunology Expert Committee of the Cross-Strait Medical and Health Exchange Association  2020 [38]  Diagnosis and treatment of OA | 19-page document including: background, methods, recommendations | Professional society | To develop an evidence-based diagnosis and treatment guideline for OA in China based on emerging new evidence. The guideline provides recommendations for the OA diagnosis, disease risks monitoring and evaluate, treatment purpose and physical, medical and surgical interventions. This guideline is intended to serve as a tool for Chinese clinicians for the best decisions-making on diagnosis and treatment of OA (p.2) | Healthcare professionals related to the diagnosis and management of OA in western medicine, integrated traditional Chinese and western medicine, and traditional Chinese medicine. Also, OA patients (p.4) | Developed through systematic review of the literature based on questions using the PICO (Population, Intervention, Comparison, and Outcome) framework. The grading of recommendations assessment, development and evaluation (GRADE) approach was used to rate the quality of evidence and the strength of recommendations, and the RIGHT (Reporting Items for Practice Guidelines in Healthcare) checklist was followed to report the guideline (p.2). Face-to-face consensus meetings and feedback comments were collected, and the expert group discussed and reviewed all recommendations and the quality of the evidence (p.5) |
| AAOS Clinical Practice Guideline Summary  Management of Osteoarthritis of the Hip  American Academy of Orthopaedic Surgeons  2020 [39]  Management of OA | 4-page document including: rationale, strength of recommendation descriptions and recommendations | Professional society | Provides guidance based on the best available evidence, to assist practitioners of all specialties, patients, and other stakeholders in deciding how to treat this common condition. Use of this guideline should help practitioners decide what interventions to try before recommending surgery and also which ones are most likely to provide cost effective benefit (p.1, p.2) | Those taking care of patients with osteoarthritis of the hip (p.1) | A group of experts with knowledge of orthopaedic surgery, physical therapy, and musculoskeletal radiology developed 18 recommendations for nonoperative and operative treatment based on relevant literature. Developing the guideline was followed using the same protocol as has been used previously for other AAOS guidelines. Once completed, peer review was requested of 21 different organizations. Of those requested, seven reviewers representing six organizations provided review. In addition, a public comment period was provided, and an additional organization provided feedback (p.1, p.2) |
| The Italian Society for Rheumatology clinical practice guidelines for the diagnosis and management of knee, hip and hand osteoarthritis  The Italian Society for Rheumatology  2019 [40]  Management of OA | 17-page document including: summary, introduction, materials and methods, recommendations, discussion, appendix | Professional society | To offer revised, evidence-based, and adapted guidelines for the diagnosis and treatment of patients with knee, hip and hand OA (both primary and secondary) in Italy (p.2) | Attending physicians and health professionals who manage patients with knee, hip and hand; patients, policy makers and those responsible for commissioning care for patients with knee, hip and hand OA in the Italian National Health Service (NHS) (p.2) | From a systematic search in databases and grey literature, 11 CPGs were selected and appraised by two independent raters. Combining evidence and statements from these CPGs and clinical expertise, 16 guidelines were developed and graded according to the level of evidence. Agreement and potential impact on clinical practice were agreed. The working group consisted of ten rheumatologists. The Guideline quality was assessed by two raters using the on-line Appraisal of Guidelines Research and Evaluation (AGREE) II instrument. An online survey from the response of the working group was considered to finalize the guidelines (p.1, p.4, p.5) |
| OARSI guidelines for the non-surgical management of knee, hip, and polyarticular osteoarthritis  Osteoarthritis Research Society International  2019 [41]  Management of OA | 12-page document including: introduction, methods, results, recommendations (knee OA, hip OA, polyarticular OA), discussion and an appendix listing Panel member | Academic group | To update and expand upon prior Osteoarthritis Research Society International (OARSI) guidelines by developing patient-focused treatment recommendations for individuals with knee, hip, and polyarticular osteoarthritis (OA) that are derived from expert consensus and based on objective review of high-quality meta-analytic data (p.1578) | Physicians other than orthopedic surgeons (p.1578) | Developed through a systematic literature review with studies being matched with PICO questions and adhering to Grading of Recommendations Assessment, Development, and Evaluation (GRADE ) methodology. This was followed by an anonymous online survey with a Voting Panel of 13 members from the fields of rheumatology, orthopedic surgery, primary care, sports medicine, physical therapy, and pharmacology (p.1579) |
| An updated algorithm recommendation for the management of knee osteoarthritis from the European Society for Clinical and Economic Aspects of Osteoporosis, Osteoarthritis and Musculoskeletal Diseases (ESCEO)  ESCEO  2019 [42]  Management and treatment of OA | 14-page document including: introduction, methods, results, discussion | Non-governmental organization | To develop an updated stepwise algorithm of recommendations in order to provide practical, current guidance that will enable clinicians to deliver patient-centric care in osteoarthritis practice (p.338) | Clinicians (p. 338) | Using the Grading of Recommendations Assessment, Development and Evaluation (GRADE) process, a summary of evidence document for each intervention in osteoarthritis was provided to all members of a European Society for Clinical and Economic Aspects of Osteoporosis, Osteoarthritis and Musculoskeletal Diseases working group, who were required to evaluate and vote on the strength of recommendation for each intervention. International working group consisted of 18 members comprising rheumatologists, specialists in physical medicine and rehabilitation, clinical epidemiologists, endocrinologists, pharmacologists, orthopedic surgeons, geriatricians, specialists in public health and health economics, research scientists and patient representatives all of whom are experienced in the performance, analysis and interpretation of clinical trial evidence related to OA (p. 338) Final algorithm was constructed based on evidence collected, and on the strength of recommendations afforded by consensus of the working group (p.339) |
| 2018 update of the EULAR recommendations for the  management of hand osteoarthritis  European Alliance of Associations for Rheumatology (EULAR)  2019 [43]  Management of OA | 9-page document including: introduction, methods, results, discussion | Non-governmental organization | To update EULAR recommendations for the management of hand OA that were published in 2007. The presented EULAR recommendations provide up-to-date guidance on the management of hand OA, based on expert opinion and research evidence (p.16). These recommendations aim to inform patients about their disease to support shared decision-making (p.22) | All health professionals who care for patients with hand osteoarthritis, patients, students, pharmaceutical industry, policy makers and health insurance companies (p.22) | A systematic literature review was performed and based on the evidence and expert opinion from an international task force of 19 physicians, healthcare professionals and patients from 10 European countries, overarching principles and recommendations were formulated. The task force further comprised 10 rheumatologists, 1 plastic surgeon, 3 healthcare professionals in the field of physiotherapy and occupational therapy and 2 patient research partners. The development of the update was performed according to the process set out in Appraisal of Guidelines for Research & Evaluation II (AGREE II) was followed. The task force formulated recommendations based on the literature and reached proposed formulations through discussion, online survey and group voting (p.14-p.17) |
| EULAR recommendations for the health professional's approach to pain management in inflammatory arthritis and osteoarthritis  European Alliance of Associations for Rheumatology (EULAR)  2018 [44]  Pain management in OA | 11-page report including: abstract, introduction, methods, results, and discussion | Non-governmental organization | To evaluate existing scientific evidence associated with the benefits of the health professional’s approach to pain management for people with inflammatory arthritis and osteoarthritis, and to use this evidence and expert opinion to provide recommendations that enable health professionals to provide knowledgeable pain-management support (p.797) | Health professionals, including rheumatologists (p.798)  Also, patients with OA and patients with the following types of inflammatory arthritis: RA, spondyloarthritis and psoriatic arthritis (p.798) | A multidisciplinary task force including professionals and patient representatives conducted a systematic literature review of systematic reviews to evaluate evidence regarding effects on pain of multiple treatment modalities. Overarching principles and recommendations regarding assessment and pain treatment were specified on the basis of reviewed evidence and expert opinion (p.797) |
| 2018 EULAR recommendations for physical activity in people with inflammatory arthritis and osteoarthritis  European Alliance of Associations for Rheumatology (EULAR)  2018 [45]  Management of OA | 10-page report including: abstract, introduction, methods, results and discussion | Non-governmental organization | To develop evidence-based recommendations for advice and guidance on physical activity in clinical practice (p.1251) | Health care professionals, patient organisations and policy makers (p.1253) | A task force (TF) (including rheumatologists, other medical specialists and physicians, health professionals, patient representatives, methodologists) from 16 countries met twice. In the first TF meeting, 13 research questions to support a systematic literature review (SLR) were identified and defined. The research evidence was categorised according to the Oxford levels of evidence. During the second TF meeting, the results from the SLR were presented, and the experts developed ‘overarching principles’ (background statements to preface recommendations) and drafted 10 recommendations through an iterative process of discussion and consensus. The recommendations were collated and sent to the TF members by email, to rate the level of agreement (LoA) independently and anonymously on a 0–10 point scale (0=totally disagree, 10=totally agree) (p.1253) |
| The Ottawa panel clinical practice guidelines for the management of knee osteoarthritis. Part one:  introduction, and mind-body exercise programs  The Ottawa Panel  2017 [46]  Management of OA | 14-page document including: introduction, methods, evaluation of existing evidence, results, discussion, limitations | Academic group | To identify effective mind-body exercise programs and provide both healthcare professionals and knee osteoarthritis patients with updated, high-quality recommendations supporting non-traditional land-based exercises for knee osteoarthritis (p.584) | Clinicians and patients (p.583) | A systematic search and adapted selection criteria included comparative controlled trials with mind-body exercise programs for patients with knee osteoarthritis. A panel of experts reached consensus on the recommendations using a Delphi survey. A hierarchical alphabetical grading system was based on statistical significance and clinical importance. All experts (Experts Panel composed of health professionals (i.e., clinicians, researchers and a patient with knee osteoarthritis) completed an online Delphi questionnaire to endorse and determine whether the draft guidelines were in agreement with the recommendations, if they were comprehensible and appropriate for the target population, and whether the literature search was applicable and thoroughly related to mind-body, strengthening and aerobic exercises in the management of knee osteoarthritis (p.583,584,586) |
| The Ottawa panel clinical practice guidelines for the management of knee osteoarthritis. Part two:  strengthening exercise programs  The Ottawa Panel  2017 [47]  Management of OA | 16-page document including: introduction, methods, results, discussion, comparisons with previous clinical practice guidelines, limitations | Academic group | To identify effective strengthening exercise programs and provide both healthcare professionals and knee osteoarthritis patients with updated, high-quality recommendations supporting traditional land-based exercises for knee osteoarthritis (p.597) | Healthcare professionals and knee osteoarthritis patients (p.597) | A systematic search and adapted selection criteria included comparative controlled trials with strengthening exercise programs for patients with knee osteoarthritis. A panel of experts reached consensus on the recommendations using a Delphi survey. A hierarchical alphabetical grading system was based on statistical significance and clinical importance (p.597). Further details can be found in Brosseau et al. (p.598) |
| The Ottawa panel clinical practice guidelines for the management of knee osteoarthritis. Part three:  aerobic exercise programs  The Ottawa Panel  2017 [48]  Management of OA | 13-page document including: introduction, methods, results, discussion, comparisons with previous clinical practice guidelines, physiological effects of therapeutic exercises, limitations | Academic group | To identify effective aerobic exercise programs and provide clinicians and patients with updated, high-quality recommendations concerning traditional land-based exercises for knee osteoarthritis (p.613) | Healthcare professionals and individuals with knee osteoarthritis (p.620) | A systematic search and adapted selection criteria included comparative controlled trials with strengthening exercise programs for patients with knee osteoarthritis. A panel of experts reached consensus on the recommendations using a Delphi survey. A hierarchical alphabetical grading system was used, based on statistical significance and clinical importance improvement) (p.613). Further details on the methodology used for this systematic review, comprised of five steps evaluating the existing evidence followed by the creation of the recommendations, and can be found in Brosseau et al. (p.614) |
| Hip pain and mobility deficits--hip osteoarthritis: Revision 2017 clinical practice guidelines linked to the international classification of functioning, disability, and health from the orthopaedic section of the American Physical Therapy Association.  American Physical Therapy Association  2017 [49]  Hip osteoarthritis, hip pain, mobility deficits | 37-page document including: summary of recommendations, introduction, methods, clinical guidelines (impairment/function based diagnosis, examination, and interventions), author/reviewer affiliations and contacts, and 7 appendices on search strategies, search results, inclusion and exclusion criteria, flow chart of articles, articles included in recommendations by topic, levels of evidence table, and procedures for assigning levels of evidence. | Professional society | To review recent peer-reviewed literature and make recommendations related to hip pain and mobility deficits (p.4) | Clinicians, patients  Also for educators, payers, policy makers, and researchers (p.6) | Content experts were appointed by the Orthopaedic Section of the American Physical Therapy Association to conduct a review of the literature and to develop an updated hip osteoarthritis (OA) clinical practice guideline as indicated by the current state of the evidence in the field. The authors worked with research librarians with expertise in systematic review to perform a systematic search for hip OA articles published since 2008. Individual clinical research articles were graded according to criteria adapted from the Centre for Evidence-based Medicine (Oxford, UK) for diagnostic, prospective, and therapeutic studies. The strength of the evidence supporting the recommendations was graded. The drafted guideline was reviewed for validation by expert reviewers, the public, a panel of consumer/patient representatives and external stakeholders, such as claims reviewers, medical coding experts, academic educators, clinical educators, physician specialists, and researchers (p.4-6) |
| EULAR recommendations for the use of imaging in the clinical management of peripheral joint osteoarthritis  European Alliance of Associations for Rheumatology (EULAR)  2017 [50]  Management of OA | 11-page report including: abstract, introduction, methods, results, discussion. | Non-governmental organization | To develop evidence-based recommendations for the use of imaging in the clinical management of the most common arthropathy, osteoarthritis (p.1484) | Clinicians who treat OA in their clinical practice (p. 1484) | A task force (including rheumatologists, radiologists, methodologists, primary care doctors and patients) from nine countries defined 10 questions on the role of imaging in OA to support a systematic literature review. The results of the literature review were presented and the experts of the Task Force developed ‘over-arching’ statements and drafted seven recommendations through a process of discussion and consensus. The Task Force confirmed the final wording of the recommendations and scored the perceived level of agreement for each statement using a 0–10 numeric rating scale (0=fully disagree; 10=fully agree), reflecting both literature evidence and expert opinion (p.1484-1485) |
| 2017 update of the Turkish League Against Rheumatism (TLAR) evidence-based recommendations for the management of knee  osteoarthritis  Turkish League Against Rheumatism  2017 [51]  Management of OA | 17-page document including: introduction, materials and methods, results, recommendations, and discussion | Academic group | To update 2012 TLAR recommendations for management of knee OA in line with developing medical knowledge and scientific evidence. To provide scientific guidance for health policy makers and health (p.1327) | Physicians and other health professionals, patients with knee OA (p.1327) | Prepared by a committee of 23 physicians with 22 physical medicine and rehabilitation (PM&R) specialists and an orthopaedic surgeon. An initial systematic literature search was performed, and articles were assessed for quality and classified according to hierarchy for the level of evidence. The selected ones were sent to committee members electronically and voting was electronically done using a visual analogue scale (p.1316) |
| PANLAR Consensus Recommendations for the Management in Osteoarthritis of Hand, Hip, and Knee  Pan-American League of Associations for Rheumatology (PANLAR)  2016 [52]  Management of OA | 10-page report including: methods, discussion, and conclusion | Professional society | To update the recommendations for the treatment of hand, hip, and knee osteoarthritis (OA) by agreeing on key propositions relating to the management of hand, hip, and knee OA, by identifying and critically appraising research evidence for the effectiveness of the treatments and by generating recommendations (p.345) | Healthcare providers involved in the management of patients with hand, hip, and knee OA (p.350) | Recommendations were developed by a group of 48 specialists of rheumatologists, members of other medical disciplines (orthopedics and physiatrists), and three patients, one for each location of OA. A systematic review of existing articles, meta-analyses, and guidelines for the management of hand, hip, and knee OA was undertaken. The scores for Level of Evidence and Grade of Recommendation were proposed and fully consented within the committee based on The American Heart Association Evidence-Based Scoring System. Two sessions were conducted with the aim of reaching agreement on the final recommendations for OA. Consensus was reached using a variation of the Delphi technique (p.345) |
| Ottawa Panel evidence-based clinical practice guidelines for therapeutic exercise in the  management of hip osteoarthritis  The Ottawa Panel  2016 [53]  Management of OA | 12-page document including: target populations, introduction, methods, results, discussion, limitations, conclusions | Academic group | To identify effective land-based therapeutic exercise interventions and provide evidence-based recommendations for managing hip osteoarthritis.  To develop an Ottawa Panel evidence-based clinical practice guideline for hip osteoarthritis (p.935) | Healthcare professionals and clinicians; may also benefit those interested in managing their hip osteoarthritis through non-pharmacological  methods (p.936) | The search strategy and modified selection criteria from a Cochrane review were used. An Expert Panel arrived at a Delphi survey consensus to endorse the recommendations. The Ottawa Panel hierarchical alphabetical grading system considered the study design according to statistical significance and clinical importance. (p.935) The Ottawa Panel develops evidence-based clinical practice guidelines using: (1) a systematic review following Cochrane Collaboration methodology; (2) calculations of the clinical importance of an intervention based on the minimal clinically important difference of common validated osteoarthritis outcomes; (3) the Ottawa Panel grading system for recommendations; and (4) an Expert Panel of health professionals who review and approve the final guideline recommendations (p.937) |
| Surgical Management of Osteoarthritis of the Knee  American Academy of Orthopaedic Surgeons  2015 [54]  Management of OA | 669-page document including: a summary of recommendations, introduction, methods, recommendations (total knee arthroplasty, patient-specific technology, and post-operative mobilization), and 12 appendices | Professional society | To develop a guideline on the surgical management of knee OA for improving treatment based on the current best evidence and serving an educational tool to guide qualified physicians through a series of treatment decisions aimed at improving the quality and efficiency of care (p.27) | Orthopedic surgeons and physicians managing adult patients with OA of the knee, healthcare professionals involved in adult OA care, decision makers and developers of practice guidelines (p.27) | Prepared by the AAOS Surgical Management of Osteoarthritis of the Knee guideline physician guideline development group (clinical experts) with the assistance of the AAOS Evidence-Based Medicine Unit in the Department of Research and Scientific Affairs (methodologists) at the AAOS. The guideline development group defined the scope of the guideline by creating PICO Questions. Upon completion of the systematic reviews, the physician guideline development group participated in a three-day recommendation meeting during which they evaluated and integrated all material to develop the final recommendations. The draft guideline recommendations and rationales received final review by the methodologists to ensure that they were consistent with the data (p.30) |
| EULAR recommendations for the non-pharmacological core management of hip and knee osteoarthritis  European Alliance of Associations for Rheumatology (EULAR)    2013 [55]  Management of OA | 11-page document including: abstract, introduction, methods, results, and discussion. | Non-governmental organization | To develop evidence-based recommendations and a research and educational agenda for the non-pharmacological management of hip and knee osteoarthritis (OA) (p.1125) | All healthcare providers involved in the delivery of non-pharmacological interventions, researchers in the field of OA, officials in healthcare governance, reimbursement agencies and policy makers. Also for people with hip or knee OA (p.1125) | The multidisciplinary task force comprised 21 experts: nurses, occupational therapists, physiotherapists, rheumatologists, orthopaedic surgeons, general practitioner, psychologist, dietician, clinical epidemiologist and patient representatives. After a preliminary literature review, a first task force meeting and five Delphi rounds, provisional recommendations were formulated in order to perform a systematic review. A literature search of Medline and eight other databases was performed up to February 2012. Evidence was graded in categories I–IV and agreement with the recommendations was determined through scores from 0 (total disagreement) to 10 (total agreement) (p.1125) |
| Ottawa Panel Evidence-Based Clinical Practice Guidelines for Aerobic Walking Programs in the Management of Osteoarthritis  The Ottawa Panel  2012 [56]  Management of OA | 17-page document including: methods, results, discussion, limitation, conclusion/clinical implications, Appendix 1-4 (Evidence-Based Clinical Practice Guidelines related to aerobic walking program interventions for the management of OA of the knee; literature search strategy; summary of the included studies; flow diagram) | Academic group | To update the Evidence-Based Clinical Practice Guidelines (EBCPGs) on aerobic walking programs for the management of osteoarthritis (OA) of the knee (p.1269)  To create an Evidence-Based Clinical Practice Guidelines for an aerobic walking program in the management of OA of the knee, in order to support health professionals and their patients diagnosed with OA in choosing the most effective aerobic walking programs for this population (p.1270) | Clinicians and researchers (p.1270) | The methodology of this project followed the Preferred Reporting Items for Systematic and Meta-Analyses checklist from the Journal of the American Physical Therapy Association, the Ottawa Expert Panel methods, and used a quantitative grading system. In conjunction with the methodology of previous Ottawa Panel publications, the construction of the guideline was developed using the Appraisal of Guidelines Research and Evaluation criteria. Following this, the Ottawa Panel individual recommendations were graded based on the strength of evidence (Table 1) (p.1270) |
| Physiotherapy in hip and knee osteoarthritis: development of a practice guideline concerning initial assessment, treatment and evaluation  Pan-American League of Associations for Rheumatology (PANLAR)  2011 [57]  Assessment, treatment, and evaluation of OA | 15-page report including: introduction, methods, results, and discussion | Professional society | To describe evidence-based physiotherapy for hip and knee osteoarthritis, including initial assessment, interventions, and assessment of outcome, based on the International Classification of Functioning, Disability and Health (p.269) | Not reported | A guideline steering committee, comprising 10 expert physiotherapists, selected topics concerning the guideline chapters: initial assessment, treatment and evaluation. A systematic literature search was performed using various databases, and the evidence was graded according to the Evidence Based Recommendation Development. By means of five consensus meetings and eight feedback rounds of the Guideline Steering Committee, recommendations were formulated and their strength graded A–D, based on the category of efficacy evidence. A first draft of the guideline was reviewed by 17 experts from different professional backgrounds. A second draft was field-tested by 45 physiotherapists (p.268-269) |
| Ottawa Panel Evidence-Based Clinical Practice Guidelines for the Management of Osteoarthritis in Adults Who Are Obese or Overweight  The Ottawa Panel  2011 [58]  Management of OA | 19-page document including: background and purpose, method, results, discussion, implications for practice, appendix | Academic group | To construct an updated evidence-based clinical practice guideline on the use of physical activity and diet for the management of osteoarthritis (OA) in adults (>18 years of age) who are obese or overweight (body mass index >25 kg/m2) (p.1) | Not reported | An a priori literature search was conducted for articles related to obesity and OA of the lower extremities that were published from January 1, 1966, to November 30, 2010. Inclusion criteria and the methods to grade the recommendations were created by the Ottawa Panel. Recommendations were graded based on the strength of evidence as well as experimental design. In agreement with previous Ottawa Panel methods, Cochrane Collaboration methods were utilized for statistical analysis. The Ottawa Panel and the research assistance team ultimately agreed on the final articles according to the inclusion and exclusion criteria (p.1, p.5) |
| Ottawa panel evidence-based clinical practice guidelines for patient education programmes in the management of osteoarthritis  The Ottawa Panel  2011 [59]  Patient education in OA | 41-page document including: introduction, methods, results, discussion, limitations, implications for practice, Appendix A, Appendix B | Academic group | To develop guidelines and recommendations on patient education programmes of any type, targeted specially to individuals with OA and which were designed to improve the clinical effectiveness of managing OA (p.320) | Not reported | The Ottawa Methods Group is made up of 13 expert methodologists with extensive experience and qualifications in developing EBCPGs. The Ottawa Methods Group contacted specialized organizations that focus on management for individuals diagnosed with OA to nominate nine experienced clinicians specializing in various fields – rheumatology, physiatry, medicine, occupational therapy, physical therapy – and a patient with OA. The EBCPGs in this report were created based on the Appraisal of Guidelines Research and Evaluation (AGREE) criteria. The Ottawa Panel graded the recommendations according to levels, and strength of evidence (p.319-320) |
| EULAR evidence-based recommendations for the diagnosis of knee osteoarthritis  European Alliance of Associations for Rheumatology (EULAR)  2010 [60]  Diagnosis of OA | 7-page report including: introduction, methods, results, and discussion. | Non-governmental organization | To develop evidence-based recommendations for diagnosis of knee OA (p.483) | Any health professional who is involved with the diagnosis of knee OA (p.483) | A multidisciplinary guideline development group, comprising 17 OA experts from 12 European countries, was commissioned by the European Alliance of Associations for Rheumatology (EULAR) Standing Committee for Clinical Affairs. After a single face-to-face meeting, each participant independently submitted up to 10 propositions related to key aspects in the diagnosis of knee OA. Consensus was reached using the Delphi technique. To develop recommendations for diagnosis, a systematic search of the literature published between January 1950 and January 2008 was undertaken. Secondary analyses were undertaken to test directly the recommendations using multiple predictive models in two populations from the UK and the Netherlands. Strength of recommendation (SOR) was graded using the EULAR 0–100 mm visual analogue scale (p.483) |
| Clinical practice guidelines for rest orthosis, knee sleeves, and unloading knee braces in knee osteoarthritis  The French Physical Medicine and Rehabilitation Society (SOFMER)  2009 [61]  Bracing in OA | 8-page document including: introduction, methods, results, discussion, recommendations, limits, Appendix A (supplementary data) | Academic group | To develop clinical practice guidelines concerning the use of bracing – rest orthosis, knee sleeves and unloading knee braces – for knee osteoarthritis (p.629) | Not reported | The French Physical Medicine and Rehabilitation Society (SOFMER) methodology, associating a systematic literature review, collection of everyday clinical practice, and external review by multidisciplinary expert panel, was used. (p.629). This three-stage method, involving a systematic review of the literature, an analysis of professional practices and final scientific committee advice, has been previously described (p.630) |
| Usefulness of taping in lower limb osteoarthritis. French clinical practice guidelines  The French Physical Medicine and Rehabilitation Society (SOFMER)  2008 [62]  Lower limb OA | 4-page document including: introduction, methods, results, discussion, recommendations | Academic group | To develop clinical practice guidelines about the use of taping in the management of lower limb osteoarthritis (p.475) | Not reported | We used the methodology advocated by the SOFMER, which combines a literature review, collection of data on current practice patterns, and validation of the recommendations by a multidisciplinary panel of experts (p.475) |
| Is there an evidence-based efficacy for the use of foot orthotics in knee  and hip osteoarthritis? Elaboration of French clinical practice guidelines  The French Physical Medicine and Rehabilitation Society (SOFMER)  2008 [63]  Foot orthotics in OA | 7-page document including: introduction, methods, results, professional practice collection, discussion, clinical practice guidelines, perspectives | Academic group | To develop clinical practice guidelines for the use of foot orthotics (FO) in the treatment of knee and hip osteoarthritis (p.714) | Not reported | The SOFMER methodology, associating a systematic review of the literature, input from every day clinical practice and external review by a multidisciplinary expert committee, was used. (p.714). This three-stage method using a systematic review of the literature, an in-depth analysis of professional practices and final scientific committee advice, has been previously described (p.715) |
| The value of individual or collective group exercise programs for knee  or hip osteoarthritis. Elaboration of French clinical practice guidelines  The French Physical Medicine and Rehabilitation Society (SOFMER)  2007 [64]  Physical exercise in OA | 6-page document including: introduction, materials and methods, results, discussion, recommendations | Academic group | To develop clinical practice guidelines concerning individual and group exercise therapy for knee and/or hip osteoarthritis (OA) (p.741) | Not reported | We used the SOFMER methodology, combining systematic literature review, collection of everyday clinical practice, and external review by a multidisciplinary expert panel, to develop the guidelines. (p.741). The SOFMER 3-stage method for developing guidelines involves systematic literature review, collection of information about professional practice and final scientific committee review (p.743) |
| Physical exercise supervised or not by a physiotherapist in the treatment of lower-limb osteoarthritis. Elaboration of French clinical practice guidelines  The French Physical Medicine and Rehabilitation Society (SOFMER)  2007 [65]  Physical exercise in OA | 10-page document including: introduction, method, results, discussion, recommendations | Academic group | To develop clinical practice guidelines concerning supervised or unsupervised kinesiotherapy for treating lower-limb osteoarthritis (OA) (p.759) | Not reported | The SOFMER methodology, associating systematic literature review, collection of everyday clinical practice, and external review by a multidisciplinary expert panel, was used to develop guidelines (p.759) |
| Could preoperative rehabilitation modify postoperative outcomes after total hip and knee arthroplasty? Elaboration of French clinical practice guidelines  The French Physical Medicine and Rehabilitation Society (SOFMER)  2007 [66]  Management and rehabilitation in OA | 9-page report including: introduction, methods, results, discussion, limits, and clinical practice guidelines | Academic group | To develop clinical practice guidelines concerning preoperative rehabilitation for hip and knee total arthroplasty (p.189) | Not reported | Used the French Physical Medicine and Rehabilitation Society methodology, combining systematic literature review, collection of everyday clinical practice, and external review by a multidisciplinary expert panel, to develop guidelines (p. 189). The quality of each manuscript was assessed according to the grading scale of the French Agency for Accreditation and Evaluation in Healthcare (p.191) |
| Evidence-based recommendations for the role of exercise in the management of osteoarthritis of the hip or knee - The MOVE consensus  British Society for Rheumatology  2005 [67]  Management and treatment of OA | 7-page document including: methods, results, and discussion | Academic group | To produce evidence-based recommendations to guide health-care practitioners concerning the role of exercise therapy in the management of hip and knee osteoarthritis (p.67) | Health-care practitioners (p.67) | A multidisciplinary guideline development group was formed from representatives of professional bodies to which OA is of relevance and other interested parties. Each participant contributed up to 10 propositions describing key clinical points regarding exercise therapy for OA of the hip or knee. Ten final recommendations were agreed by the Delphi process. The research evidence for each was determined. A literature search was undertaken in the Medline, PubMed, EMBASE, PEDro, CINAHL and Cochrane databases. The methodological quality of each retrieved publication was assessed. Outcome data were abstracted and effect sizes calculated. The evidence for each recommendation was assessed and expert consensus highlighted by the allocation of two categories: (1) strength of evidence and (2) strength of recommendation (p.67) |
| EULAR evidence-based recommendations for the management of hip osteoarthritis: report of a task force of the EULAR Standing Committee for International Clinical Studies Including Therapeutics (ESCISIT)  European Alliance of Associations for Rheumatology (EULAR)  2005 [68]  Management of OA | 15-page report including: methods, results, and discussion. | Non-governmental organization | To develop evidence-based recommendations for the management of hip osteoarthritis (p.669) | Healthcare provider groups (p.679) | The multidisciplinary guideline development group comprised 18 rheumatologists, 4 orthopaedic surgeons, and 1 epidemiologist, representing 14 European countries. Ten final recommendations were agreed using a Delphi consensus approach. Medline, Embase, CINAHL, Cochrane Library, and HTA reports were searched systematically to obtain research evidence for each proposition. Where possible, outcome data for efficacy, adverse effects, and cost effectiveness were abstracted. Effect size, rate ratio, number needed to treat, and incremental cost effectiveness ratio were calculated. The quality of evidence was categorised according to the evidence hierarchy. The strength of recommendation was assessed using the traditional A–D grading scale and a visual analogue scale (p.669) |
| EULAR Recommendations 2003: an evidence based approach to the management of knee osteoarthritis: Report of a Task Force of the Standing Committee for International Clinical Studies Including Therapeutic Trials (ESCISIT)  European Alliance of Associations for Rheumatology (EULAR)  2003 [69]  Management and treatment of OA | 11-page report including: methods, results, and discussion. | Non-governmental organization | To update the EULAR recommendations for management of knee osteoarthritis (OA) by an evidence-based medicine and expert opinion approach (p.1145) | Not reported | The members of the expert committee on knee OA reconvened in November 2001 to establish the methodology in updating the evidence base and recommendations for the treatment of knee OA. Databases Medline OVID and BIDS Embase were searched systematically to prepare an evidence-based review. The level of evidence found for each treatment was documented. Quality scores were determined for each paper, and an effect size comparing the treatment with placebo was calculated, where possible. The strength of recommendation for an intervention was graded. 10 recommendations were proposed and a further set of 10 items was identified for future research following a five stage Delphi technique. The committee ranked the potential toxicity of each intervention (p.1146-1147) |
