# Supplementary material for: Identifying strategies that support equitable person-centred osteoarthritis care for diverse women: content analysis of guidelines
Source: BMC Musculoskelet Disord. 2023 Sep 14;24:734. doi: 10.1186/s12891-023-06877-x (PMC10500823; doi:10.1186/s12891-023-06877-x)
Supplement: Supplementary file 4 — Additional File 4. Data extracted from included guidelines on person-centred care [file 12891_2023_6877_MOESM4_ESM.docx]

**Additional File 4. Data extracted from included guidelines on person-centred care**

| Guideline  Year [Reference] | Person-centred care domains | | | | | | Domains addressed  (n) |
| --- | --- | --- | --- | --- | --- | --- | --- |
|  | Foster healing relationship | Exchange information | Respond to emotions | Manage uncertainty | Share decisions | Enable self-management |  |
| American Academy of Orthopedic Surgeons  2021 [9] | -- | -- | -- | -- | The use of a topical gel for osteoarthritis of the knee should be an acceptable method of treatment if cost is not prohibitive and the patient does not have any skin irritation from the gel (p.31) | Self-management programs are recommended to improve pain and function for patients with knee osteoarthritis (p.8)  Patient education programs are recommended to improve pain in patients with knee  osteoarthritis (p.37) | 2 |
| American College of Rheumatology/Arthritis Foundation  2020 [10] | -- | Each patient should be assessed for the presence of medical conditions, such as hypertension, cardiovascular disease, heart failure, gastrointestinal bleeding risk, chronic kidney disease, or other comorbidities, that might have an impact on their risk of side effects from certain pharmacologic agents, as well as injuries, disease severity, surgical history, and access to and availability of services (transportation, distance, ability to take time off work, cost, insurance coverage) that might have an impact on the choice of physical, psychological, and mind-body approaches (p.152) | Patients may experience a variety of additional symptoms as a result of the pain and functional limitations arising from OA and/or comorbidities, such as mood disorders, such as depression and anxiety, altered sleep, chronic widespread pain, and impaired coping skills. The broader impact of OA on these comorbidities is of particular importance when choosing among treatment options (p.153)  Measures aimed at improving mood, reducing stress, addressing insomnia, managing weight, and enhancing fitness may improve the patient’s overall well-being and OA treatment success (p.153) | -- | There is no uniformly accepted level of pain at which a patient should or should not exercise, and common-sense approach of shared decision-making between the treating clinician and the patient regarding when to initiate an exercise training is advisable (p.154)  The use of centrally acting agents such as duloxetine and the use of hyaluronic acid injections should be based on shared decision-making between the physician and patient (p.158)  Treatment decisions should take the personal beliefs and preferences of the patient into account (p.152)  Conditional recommendations are those for which the majority of informed patients would choose to follow the recommended course of action, but some would not. They are value- and preference-sensitive and always warrant a full shared decision-making approach involving a complete and clear explication of benefits, harms, and burdens in language and in a context that patients understand (p.152)  Individual preferences, access, and affordability are likely to play a role in what works best for an individual patient (p.154)  The choice of any single or group of interventions may vary over the course of the disease or with patient and provider preferences, and is optimally arrived at through shared decision-making (p.160) | Self-efficacy and self-management programs are strongly recommended for patients with knee, hip, and/or hand OA (p.154)  A comprehensive plan for the management of OA in an individual patient may include educational, behavioral, psychosocial, and physical interventions (p.153)  Exercise programs are more effective when combined with self-efficacy and self-management interventions or weight-loss programs (p.154)  Self-efficacy and self-management programs use a multidisciplinary group–based format combining sessions on skill-building (goal-setting, problem-solving, positive thinking), education about the disease and about medication effects and side effects, joint protection measures, and fitness and exercise goals and approaches (p.154) | 4 |
| American Physical Therapy Association  2020 [36] | -- | -- | -- | -- | Supervised physical therapist management should be provided for patients who have undergone total knee arthroplasty. The optimal setting should be determined by patient safety, mobility, and environmental and personal factors. (p.1606, p.1621) | Physical therapists should teach and encourage patients to  implement passive, active assistive, and active ROM exercises for the involved knee following total knee arthroplasty (p.1606,p.1615)  Physical therapists should teach patients and other care givers use of cryotherapy [while considering barriers such as cost] and encourage its use for early postoperative pain management for patients who have undergone total knee arthroplasty (p.1606)  Physical therapists or other team members should provide  preoperative education for patients undergoing total knee arthroplasty, including, at a minimum: patient expectations during hospitalization and factors influencing discharge planning and disposition, the postoperative rehabilitation program, safe transferring techniques, use of assistive devices, and fall prevention. Patient education is an essential part of patient care in all settings, particularly in an increasingly patient-centered health care environment. (p.1606,1610)  Physical therapists should develop an early mobility plan and teach patients who have undergone TOTAL KNEE ARTHROPLASTY regarding the importance of early mobility and appropriate progression of physical activity, based on safety, functional tolerance, and physiological response (p.1606,1613)  Physical therapists should design, implement, teach, and progress patients who have undergone TOTAL KNEE ARTHROPLASTY in high-intensity strength training and exercise programs during the early postacute period (i.e., within 7 days after surgery) to improve function, strength, and range of motion (p.1617)  Physical therapists should provide guidance to the care team and patient on safe and objective discharge planning, patient functional status, assistance equipment, and services needed to support a safe discharge from the acute care setting (p.1623) | 2 |
| American Academy of Orthopaedic Surgeons  2020 [37] | -- | -- | -- | -- | Risks and benefits of [Providing non-arthroplasty surgical options] must be discussed with the patients to determine who would best be suited for these types of joint preserving options (p.50)  Engagement of the surgeon and each individual patient is required to determine if the patient is a suitable candidate for same day discharge without an undue increase in peri-operative risks (p.63) | -- | 1 |
| Rheumatology and Immunology Expert Committee of the Cross-Strait Medical and Health Exchange Association  2020 [38] | -- | -- | -- | -- | -- | It is recommended to carry out health education for OA patients, mainly to educate them about the causes, prevention, progress and treatment of the disease, reduce the burden of patients’ thoughts, and improve their self-management efficiency (p.6) | `1 |
| American Academy of Orthopaedic Surgeons  2020 [39] | -- | -- | -- | -- | Practitioners should generally follow a moderate [strength] recommendation but remain alert to new information and be sensitive to patient preferences (p.2-4)  Patient preference should have a substantial influencing role on recommendations labeled as limited (p.3,4) | -- | 1 |
| The Italian Society for Rheumatology  2019 [40] | -- | Before establishing the exercise plan, it is essential to evaluate the ability to perform activities of daily living (ADLs), instruct in joint protection techniques and provide assistive devices, as needed (p.13) | -- | -- | Optimal management of OA requires a combination of non-pharmacological and pharmacological treatment modalities  individualized to the patient’s needs (p.6,9)  Treatment of hand, hip and knee OA should be individualized according to the wishes and expectations of the individual (p.9) | Information, education and an individually tailored program, including long-term and short-term goals, intervention or  action plans to reduce the degenerative damage of the OA should be provided. People with hip and/or knee OA should be taught a regular individualized (daily) exercise regimen and participate in self-management programs, strengthening,  low-impact aerobic exercises, and neuromuscular education (p.7,12)  Before establishing the exercise plan, it is essential to evaluate the ability to perform activities of daily living (ADLs), instruct in joint protection techniques and provide assistive devices, as needed. Weight loss through aerobic activity in water or not is to be set for overweight or obese subjects. The programs must be flexible and easily modifiable according to the patient’s needs and to the objectives that he/she actually achieves over time (p.17)  Patients with hip and knee OA, who are overweight, should be encouraged to lose weight and maintain their weight at a lower level (p.16) | 3 |
| Osteoarthritis Research Society International  2019 [41] | -- | Factors assessed at the initial visit should include clinical, emotional, and environmental factors (environmental factors are sleep quality, social networks, health beliefs and expectations, mood) (p.1585, Fig 2) | -- | Clinicians are encouraged to continually provide their patients with necessary information about OA disease progression and self-care techniques and to promote hope, optimism, and a positive expectation of benefit from treatment (p.1583) | During the initial  assessment, clinicians select core Treatment(s) tailored to individual needs and preferences (p.1582) | Structured land-based exercise programs, dietary weight management in combination with exercise, and mind-body exercise (such as Tai Chi and Yoga) were considered by the panel to be effective and safe for all patients with Knee OA, regardless of comorbidity. These treatments are recommended for use alone or along with interventions of any recommendation level, as deemed appropriate for the individual. Education about OA is considered a standard of care (p.1582)  Aquatic exercise, gait aids, cognitive behavioral therapy with an exercise component, and self-management programs were the recommended non-pharmacologic options for individuals with Knee OA and no comorbidities (p.1583) | 4 |
| European Society for Clinical and Economic Aspects of Osteoporosis, Osteoarthritis and Musculoskeletal Diseases (ESCEO)  2019 [42] | -- | -- | -- | -- | [Non-pharmacological treatments] should be personalized and adjusted to the characteristics of the patient and their environment (p.339) | Strong recommendation to the application of a core set comprising: information access/education, weight loss and an exercise program, which is applicable throughout the management of knee OA (p.339) | 2 |
| European Alliance of Associations for Rheumatology (EULAR)  2019 [43] | -- | -- | -- | -- | Shared decision-making, an approach to healthcare in which health professionals and patients mutually share information to reach consensus about the preferred management strategy, should be the basis of management in hand OA. This overarching principle implies that not only the best available evidence, but also the patients’ wishes and expectations are important to be considered when making decisions on managing the disease. Achieving shared decision-making depends on building and maintaining a good relationship between patient and health professional, and sharing the best evidence, in order to be able to make an informed decision. It pertains to all stages of management, including, for example, setting a treatment goal, choosing the best strategy to achieve it or considering other strategies when the treatment goal is not reached (p.18) | All patients should be offered information on the nature and course of the disease, as well as education on self-management principles and treatment options (p.18)  In patients with chronic complaints returning for follow-up, information and education provision should be an ongoing process involving reinforcement and expansion (p.18)  Long-term follow-up of patients with hand OA should be adapted to the patient’s individual needs-this includes patients’ wishes and expectations…‘Individual needs’ that may be taken into consideration when assessing the need for follow-up include severity of symptoms, presence of erosive disease, use of a pharmacological therapy that needs re-evaluation and patient’s wishes and expectations…follow-up will likely increase adherence to non-pharmacological therapies like exercise or orthoses, and provides an opportunity for re-evaluation of treatment (eg, revision of orthoses, or adjustment of pharmacological treatment) For most patients, standard radiographic follow-up is not useful at this moment. Follow-up does not necessarily have to be performed by the rheumatologist. At what moment other health professionals should refer a patient back to the rheumatologist, should be considered at an individual patient level (p.21) | 2 |
| European Alliance of Associations for Rheumatology (EULAR)  2018 [44] | -- | Invite patients disclosing the impact of pain on their daily functioning, to assess their ideas and concerns regarding the cause of their pain and the perceived control over pain episodes, and to take account of their expectations and preferences for treatment. It is deemed important to establish the patient’s functional and valued life goals, that is, what it is that they cannot currently do as well as they would wish to (p.802)  Assess sleep problems: the quantity and quality of sleep, including whether the patient feels refreshed on waking and sleep hygiene habits such as regular exercise during the day, stress management, noise, sleep timing and avoidance of caffeine, nicotine, alcohol and daytime napping (p.802)  Assess social factors related to pain and its consequences: the way family members and other significant others react to patient’s pain or pain-related disability; work; family and friends; economic problems; housing (p.802)  Assess other factors that might influence pain or pain management, such as dependence on tobacco, alcohol or drugs (p.802)  Assessment by the health professional should include the following aspects (the assessment is brief or extensive depending on factors such as available time, whether it is a first or regular consultation, and the needs of the patient:  -Patient’s needs, preferences and priorities regarding pain management and important activities, values and goals in daily life.  -Patient’s pain characteristics including severity, type, spread and quality.  -Previous and ongoing pain treatments and the perceived efficacy.  -Current inflammation and joint damage as sources of pain, and whether these are adequately treated.  -Pain-related factors that might need attention: (a) the nature and extent of pain-related disability, (b) beliefs and emotions about pain and pain-related disability, (c) social influences related to pain and its consequences, (d) sleep problems and (e) obesity (p.803)  Validation of the patient’s pain experience is considered a prerequisite for trust, communication and engagement in treatment (p.801)  Assess pain severity using a numerical or visual analogue pain rating scale, and the onset, duration, location and spread (pain manikin), quality, interference, triggers and progression of pain (p.802)  Assess the nature and extent of disability: physical activity, mobility, activities of daily living, social participation, general physical fitness (aerobic capacity, muscle strength, endurance), pain-related fear and avoidance of activities, balance of activities and rest (pacing) (p.802)  Ask about the patient’s existing use of prescribed and over-the-counter pain relief including homeopathic remedies and consider if the frequency of use is safe (not over dosing) and appropriately regular. Ask or refer for further specialist or medical advice if there are concerns or if additional pharmacological treatment may be indicated (p.803) | Assess beliefs and emotions about pain and pain-related disability: the psychological response to pain and psychological vulnerability factors, psychological distress, psychiatric comorbidity and cognitions such as catastrophizing (rumination, magnification and helplessness), fear of movement-related pain, catastrophizing and pain self-efficacy (p.802)  If there are indications that social variables or psychological factors interfere with effective pain management and functional status, then consider (depending on the severity) providing basic social and psychological management support or referral to a psychologist, social worker, self-management support programme, CBT or multidisciplinary treatment. If psychopathology (e.g., depression and anxiety) is present, discuss treatment options with the patient and the patient ’s primary care physician (p.803)  If psychosocial factors such as fear of movement or catastrophising cognitions underlie a disabled, sedentary lifestyle, then consider a multidisciplinary intervention including cognitive-behavioural therapy (p.803) | -- | Patient-centred care was considered important. Care that is respectful of and responsive to individual patient preferences, needs and values, and ensures that patient values guide clinical decisions, may improve adherence and persistence with treatment (p.801)  The health professional and patient appraise whether advice to stay active, supervised physical exercise or multidisciplinary treatment is needed (p.803)  Assess ongoing pharmacological and non-pharmacological treatments, previous treatments tried and the effects and side effects of these treatments, patient beliefs about the ability to control and overcome pain and its consequences, and willingness of the patient to engage in additional treatment if deemed necessary (p.802)  The patient should receive a personalized management plan with the aim of reducing pain and pain-related distress and improving pain-related function and participation in daily life. This plan is guided by shared decision-making, the expressed needs of the patient, the health professional’s assessment and evidence-based treatment options (p.803) | If the patient is not able to initiate physical activity and exercises without help, then consider the possibility for referral to a physiotherapist for individually tailored graded physical exercise or strength training (p.803)  If a patient has pain during activities of daily living which impedes functioning, orthotics (such as splints, braces, gloves, sleeves, insoles and shoes), daily living aids (such as a tin opener), an assistive device (such as a cane or rollator) or ergonomic adaptation (at home, workplace) can be offered. If the patients wants to use this assistive support, then consider referral to the occupational therapist, who can proceed with several actions: offer education about appropriate ways to use joints and ergonomic principles, appraise the need for the use of an orthotic or assistive device, give advice about how to acquire it, fit the customised aid to the patient, offer training in the use of it, refer to the appropriate specialist who will do this, e.g., orthopaedic shoemaker (p.803)  If sleep disturbance is reported, inquire about causes (e.g., pain, persistent worrying, poor sleep habits) and offer basic education about good sleep hygiene practices. If sleep remains (severely) disturbed, refer to a therapist or programme aimed at restoring sleep, or to a specialised sleep clinic (p.803)  If the patient is obese, explain to the patient that obesity can contribute to pain and disability. Discuss accessible weight management options with the patient or signpost appropriate specialized weight management support; for example, dietitian, psychologist, community lifestyle services or bariatric clinic/surgery (p.803) | 4 |
| European Alliance of Associations for Rheumatology (EULAR)  2018 [45] | -- | Increase health professionals and physicians’ skills in communicating the role of physical activity in managing general health and disease-specific issues (p.1256) | -- | -- | Healthcare providers should evaluate the type, intensity, frequency and duration of the people’s actual physical activity by means of standardised methods to identify which of the four domains of general physical activity recommendations can be targeted for improvement (p.1255)  The physical activity-interventions should be based on individual aims, which should be regularly evaluated. This can be done by physical activity assessments and any other assessments related to the individual aims. As physical activity assessments, performance–based tests, patient-reported outcome measures (e.g., SQUASH, PASE) and self-monitoring tools (e.g., wearables such as Fitbit, pedometer, or accelerometer) were identified (p.1256)  Healthcare providers should consider different modes of delivery of physical activity (e.g., supervised/not-supervised, individual/group, face-to-face/online, booster strategies) in line with people’s preferences (p.1255) | Healthcare providers should plan and deliver physical activity interventions that include the behavioural change techniques self-monitoring, goal setting, action planning, feedback and problem solving (p.1255)  All healthcare providers involved in the management of people with osteoarthritis should take responsibility for promoting physical activity and should cooperate, including making necessary referrals, to ensure that people with inflammatory arthritis and osteoarthritis receive appropriate physical activity interventions (p.1255)  Disease-specific facilitators included positive impact of exercise in symptoms or disease control, information about disease and correct exercising, the use medication for pain prior to exercising, using self-regulation techniques, supportive, but not controlling encouragement from health care professionals and a supportive social background (p.1256) | 3 |
| The Ottawa Panel  2017 [46] | -- | -- | -- | -- | -- | -- | 0 |
| The Ottawa Panel    2017 [47] | -- | -- | -- | -- | This guideline provides more specificity as to the components of the strengthening exercise programs and ultimately, better applicability for clinicians interested in implementing these programs based on their patients’ preferences (p.605) | A six-week osteoarthritis education and supervised strengthening exercise program with home exercises (resistance exercises for the knee and hip independently of the site of major pain) (one two-hour  session per week) for the management of adults with knee osteoarthritis for pain relief (VAS) 54 at the six-month follow-up is suggested (p.599)  A 12-week home-based physiotherapist prescribed supervised quadriceps strengthening  exercise program (using ankle weights, therapeutic elastic bands) (five days per week) for the management of  knee osteoarthritis for pain relief (WOMAC subscale) at 12 weeks end of treatment is strongly recommended (p.599)  A six-week progressive exercise program (warm-up, strengthening, balance and motor control exercises), education and usual care (45 minutes, two times per week), either in a group or individually, for the management of knee osteoarthritis for improved physical function (WOMAC subscale) at the end of treatment of six weeks is strongly recommended. (p.600)  A two-week mechanical diagnosis and therapy exercise program (end-range exercises; advice on exercises for aerobic as well strengthening of the quadriceps; biking; walking) (10 repetitions every 2–3 hours) for the management of knee osteoarthritis for pain relief (P4 subscale), pain relief (KOOS pain subscale) and improved physical function (KOOS in daily living subscale) at two weeks end of treatment is strongly recommended (p.601)  A 12-week strengthening exercise program with home exercises and usual care, including patient education and medication if necessary (exercises for muscle functions, mobility and coordination and  instructions) (one to three days per week) for the management of elderly individuals with knee osteoarthritis for  pain relief (VAS) at 12 weeks end of treatment is suggested (p.602)  This guideline recommends muscle-strengthening  exercises with or without other types of therapeutic  exercises as an effective non-pharmacological  intervention for pain relief, improved physical function and quality of life in adults with knee  osteoarthritis (p.604)  Health counselling, goal-setting, problem-solving support, telephone contacts, face-to face visits, exercise logs, social/peer support and positive feedback either alone or in various combinations, have been studied and can be adopted to promote long-term use of land-based exercises in the management of knee osteoarthritis (p.605) | 2 |
| The Ottawa Panel  2017 [48] | -- | -- | -- | -- | -- | A three-month aerobic, strengthening exercise program and osteoarthritis health education (brisk walking, isometric and isotonic muscle strengthening with therapeutic elastic bands, stretching) (one-hour sessions, three times per week) for the management of knee osteoarthritis for pain relief during weight-bearing activities (AIMS2 subscale) and improved physical function (AIMS2 subscale) at the end of treatment of three  months is strongly recommended (p.617)  10-week community physiotherapy exercise interventions (an individualized aerobic and strengthening exercise program and advice leaflet about activity and pacing) (20 minutes, three-six times over 10 weeks) for the management of knee osteoarthritis for pain relief (WOMAC subscale) and improved physical function (WOMAC subscale) at the three months follow-up is strongly recommended (p.617)  Several trials used home-based programs as an additional component with periodic health professional visits,29 telephone follow-up,28,29 as a progression component28,29 or as a concomitant intervention (p.618) | 1 |
| American Physical Therapy Association  2017 [49] | -- | Clinicians should use validated outcome measures that include domains of hip pain, body function impairment, activity limitation, and participation restriction to assess outcomes of treatment of hip osteoarthritis. Measures to assess hip pain may include the Western Ontario and McMaster Universities Osteoarthritis Index (WOMAC) pain subscale, Brief Pain Inventory (BPI), pressure pain threshold (PPT), and pain visual analog scale (VAS) (p.10)  To assess activity limitation, participation restrictions, and changes in the patient’s level of function over the episode of care, clinicians should utilize reliable and valid physical performance measures, such as the 6-minute walk test, 30-second chair stand, stair measure, timed up-and-go test, self-paced walk, timed single-leg stance, 4-square step test, and step test (p.12)  Clinicians should measure balance performance and activities that predict the risk of falls in adults with hip osteoarthritis, especially those with decreased physical function or a high risk of falls because of past history. Recommended balance tests for patients with osteoarthritis include the Berg Balance Scale, 4-square step test, and timed single-leg stance test (p.12) | -- | -- | Clinicians should individualize prescription of therapeutic activities based on patient’s values, daily life participation, and functional activity needs (p.16)  Clinicians should use individualized flexibility, strengthening, and endurance exercises to address impairments in hip range of motion, specific muscle weaknesses, and limited thigh (hip) muscle flexibility. For group-based exercise programs, effort should be made to tailor exercises to address patients’ most relevant physical impairments (p.19) | Clinicians should provide patient education combined with exercise and/or manual therapy. Education should include teaching activity modification, exercise, supporting weight reduction when overweight, and methods of unloading arthritic joints (p.16)  Clinicians should provide impairment-based functional, gait, and balance training, including the proper use of assistive devices (canes, crutches, walkers), to patients with hip osteoarthritis and activity limitations, balance impairment, and/or gait limitations when associated problems are observed and documented during the history or physical assessment of the patient (p.16)  Clinicians should collaborate with physicians, nutritionists, or dietitians to support weight reduction in individuals with hip osteoarthritis who are overweight or obese (p.20) | 3 |
| European Alliance of Associations for Rheumatology (EULAR)  2017 [50] | -- | -- | -- | -- | -- | -- | 0 |
| Turkish League Against Rheumatism (TLAR)  2017 [51] | -- | -- | -- | -- | Regarding total knee arthroplasty, the final decision should be taken by the physician and the patient together (p.1325)  Treatment should be tailored for each patient individually (p.1318)  All patients should be evaluated individually, and appropriate exercise programme should be planned accordingly (p.1319)  Knee OA patients should be informed about the natural course and goals of treatment of the disease. Appropriate life style alterations for joint protection should be advised by physician when necessary (p.1319)  While deciding total knee arthroplasty, not only the radiographic findings, but also the pain and functional status of patient should be taken into account (p.1325) | All patients with knee OA should be informed about the disease and its treatment. Patients should be educated on how they can protect their joints and how to save energy while performing occupational, sportive, daily living and recreational activities (p.1318)  All patients should be advised to perform appropriate exercises which are planned according to age, comorbidity and severity of the disease (p.1319) | 2 |
| Pan-American League of Associations for Rheumatology (PANLAR)  2016 [52] | -- | -- | -- | -- | The treatment of hand OA should be individualized according to the type of OA (nodal or erosive), its location and severity, the presence of inflammation, the pain level, the level of disability and reduction in quality of life, the comorbidities and concomitant medication, and the needs and expectations of patients (p.346)  Aerobic exercise can be implemented gradually and progressively according to each patient’s level of fitness at a frequency of three or more times per week, with a minimum duration of 20 to 30 minutes per session (p.349) | Patients with hip OA should receive information and education regarding the therapeutic objectives and the importance of changes in lifestyle, which include an exercise regimen, weight reduction, the use of walking aids (walking stick and crutches) and shoe adjustments, and other measures to prevent the progression of joint damage (p.347)  Information and education regarding treatment goals and the importance of lifestyle changes to reduce the degenerative damage of the knee joint should be provided to the patient (p.348) | 2 |
| The Ottawa Panel  2016 [53] | -- | -- | -- | -- | It is recommended that therapeutic exercise interventions be supplemented with individualized medical advice and treatment in order to ensure safety and maximal benefit (p.942) | The Ottawa Panel strongly recommends eight weeks of supervised group strength training in conjunction with  unsupervised home exercises for hip osteoarthritis  management for pain (p.941)  The Ottawa Panel strongly recommends 12 weeks of supervised group strength training, functional, and  flexibility exercises with patient education programme for hip osteoarthritis management and improvement of  physical function (WOMAC subscale) following ⩾40 weeks total (p.942) | 2 |
| American Academy of Orthopaedic Surgeons  2015 [54] | -- | The treatments and procedures for each patient relies on mutual communication between the patient, physician, and other healthcare professionals (p.4) | -- | -- | Treatment decisions should be made in light of all circumstances presented by the patient (p.4)  Knee osteoarthritis management is based on the assumption that decisions are predicated on the patient and/or the patient’s qualified heath care advocate having communication with the physician about available treatments and procedures applicable to the individual patient. Once the patient and or their advocate have been informed of available therapies and have discussed these options with his/her physician, an informed decision can be made. Clinician input based on experience with conservative management and the clinician’s surgical experience and skills increases the probability of identifying patients who will benefit from specific treatment options (p.28)  Contraindications are relative and require an in-depth discussion with the patient and physician (surgeon, anesthesiologist) about their individual risk factors. Additional factors, such as the individual’s co-morbidities, and/or specific patient characteristics may affect the physician’s choice of treatment. Clinician input based on experience increases the probability of identifying patients who will benefit from specific treatment options. The individual patient and/or their decision surrogate dynamic will also influence treatment decisions, therefore, discussion of available treatments and procedures applicable to the individual patient rely on mutual communication between the patient and/or decision surrogate and physician, weighing the potential risks and benefits for that patient. Once the patient and/or their decision surrogate have been informed of available therapies and have discussed these options with the patient’s physician, an informed decision can be made. (p.29) | Patients with symptomatic knee OA should participate in self-management (p.82) | 3 |
| European Alliance of Associations for Rheumatology (EULAR)  2013 [55] | -- | The recommendation on the initial assessment included the following elements: the person’s physical status, activities of daily living, participation, mood and health education needs, health beliefs and motivation to self-manage (p.1126) | -- | -- | The overarching principle for treatment of a person with hip or knee OA should be individualised, which is in line with previous guidelines. Individualised treatment does not imply that every treatment should be individually provided, it means rather that treatment is personalised, or tailored (p.1126)  Individualized treatment being the standard of care in OA and chronic disease in general was considered to imply informed, shared decision-making, taking into account the person’s wishes and preferences (p.1126) | Teach and encourage behavioural change strategies through goal setting of physical activity and weight changes, action plans to maintain changes and regular follow-up over at least 1 year to re-evaluate and discuss goals and action plans (p.1128)  A person with hip or knee OA should receive education about her/his condition and be managed accordingly (p.1128)  The addition of advice from a dietician for overweight or obese patients to the combination of patient education or self-management intervention plus exercise was found to improve both pain and function in patients with hip or knee OA (p.1128)  The following factors improve adherence to exercise or physical activity: individual exercise, graded activity, individualisation according to the person’s exercise goals, feedback on progress made towards the goals, iterative problem solving with emphasis on skills that will improve adherence, reinforcements of maintaining exercise such as additional motivational programmes, exercise plans and logbooks, written information and audiotape or videotape, and booster sessions (p.1128)  The content and method of delivery of various forms of educational programmes to best benefit the person with hip or knee OA. Appropriate information and education are indispensable in prompting adequate self-management in chronic diseases (p.1129)  In patients with knee OA different delivery modes (individual, group-based or home programmes) have all been shown to effectively reduce pain and improve function (p.1129)  In overweight or obese populations… elements such as self-monitoring, explicit weight-loss goals, and motivational interviewing have all been suggested to promote weight loss and that regular follow-up over 4 years helps in maintenance of the weight loss (p.1130) | 3 |
| The Ottawa Panel  2012 [56] | -- | -- | -- | -- | -- | Behavioral strategies, such as patient education, health counseling, and phone contacts, in combination with aerobic walking programs can facilitate OA management and allow individuals to increase their exercise levels (p.1275)  The most effective method in terms of long-term compliance rates consisted of a multifaceted approach that incorporated social support with aerobic walking programs (p.1276)  Aerobic walking combined with stretching and strengthening exercises, education, and/or behavior programs are recommended to improve pain relief, functional status, and QOL of adult individuals with OA (p.1274) | 1 |
| Pan-American League of Associations for Rheumatology (PANLAR)  2011 [57] | -- | A combination of questionnaires (preferably the patient specific complaint list and performance testing (preferably the timed up and go test) is recommended to use in the initial assessment and evaluating treatment goals and should have the focus on how the patient presents his complaints (p.275)  The physiotherapist should assess the patient's health status primarily in terms of activity limitations and participation restrictions. The therapist may also assess impairments of body function and structure, as well as personal and environmental factors, insofar as these relate to the limitations and restrictions (p.276) | Preoperative education could be considered if there is much anxiety for the operation (p.274) | -- | -- | In the initial assessment, patient’s main limitations and impairments are prioritized, and treatment goals and a treatment plan are formulated, and in close collaboration with the patient, treatment goals are set, with the focus on limitations of activity and restriction in participation (p.270)  Individual treatment goals should be defined. Goal setting is a shared process between the physiotherapist and the patient. Goals should be formulated according to the SMART principles (specific, measurable, achievable, realistic, and timed), for example: being able to walk 800 meters (from home to the supermarket and back) two times a week in six weeks (p. 271)  The exercise program must have a focus on limitations of activities and restrictions in participation. In some cases the exercise therapy could be adjusted to individual treatment goals. For example joint proprioception and balance training or a behavioral graded activity strategy. Decreasing the frequency of treatment sessions at the end of the treatment is needed to help the patient to achieve an independent adequate level of physical activity. To improve the transition to recreational or sport activities the patient must be guided by the physiotherapist (p.272)  Physiotherapists should provide education and promote adequate self-management in patients with osteoarthritis. Interventions should comprise the following items: knowledge and understanding of hip and knee osteoarthritis; the consequences of osteoarthritis on functions, activities and participation; the relation between the mental and physical load and carrying capacity; the way to deal with complaints caused by osteoarthritis; an active and healthy lifestyle (moving, nurturing, overweight); change in moving behavior; joint protection and the use of (walking) aids (p.272) | 3 |
| The Ottawa Panel  2011 [58] | -- | -- | -- | -- | -- | Weight loss can be facilitated by dietitians, and physical therapists play a key role in enrolling individuals who are obese or overweight in lower-extremity muscle strengthening and balance exercise programs after significant weight loss in order to maintain and develop lean muscle mass, to improve the biomechanics and stability of the knee, and to indirectly slow down the progression of knee OA. These biomechanical improvements can potentially reduce pain and enhance functional status in individuals with OA who are obese (p.851)…because physical therapists are not experts in diet, it is suggested that they work with an interdisciplinary team including dietitians (p.852)  Behavioral strategies such as patient education, health counseling, realistic and achievable goal setting, telephone contacts, 7-day physical activity and dietary logs, social/ peer support, self-management, self efficacy, and positive feedback, either alone or in numerous combinations, have been studied in populations without OA and should be explored in depth in individuals with OA who are obese or overweight (p.851) | 1 |
| The Ottawa Panel  2011 [59] | -- | -- | -- | -- | -- | Patient education is  recommended in order to improve pain relief, mobility, self-efficacy, quality of life, functional status, flexibility, psychological well-being, compliance to exercise, relaxation, and fatigue levels (p.329)  Five different education programmes were identified: pre-operative education for patients undergoing hip replacement, self-management education, joint protection and home exercise education, spousal-assisted pain coping, and education and general disease education provided through monthly telephone follow-up (p.329) | 1 |
| European Alliance of Associations for Rheumatology (EULAR)  2010 [60] | -- | -- | -- | -- | -- | -- | 0 |
| The French Physical Medicine and Rehabilitation Society (SOFMER)  2009 [61] | -- | -- | -- | -- | -- | -- | 0 |
| The French Physical Medicine and Rehabilitation Society (SOFMER)  2008 [62] | -- | -- | -- | -- | -- | -- | 0 |
| The French Physical Medicine and Rehabilitation Society (SOFMER)  2008 [63] | -- | -- | -- | -- | -- | -- | 0 |
| The French Physical Medicine and Rehabilitation Society (SOFMER)  2007 [64] | -- | -- | -- | -- | -- | -- | 0 |
| The French Physical Medicine and Rehabilitation Society (SOFMER)  2007 [65] | -- | -- | -- | -- | -- | An educational pre-program is recommended to inform the patient about the ease and effectiveness of the exercise (p.767)  Recommendation for  beginning a physical exercise program supervised by a physiotherapist and then continuing with a self-administered program at home with good compliance. The content, intensity, and frequency of the exercises must be adapted to each patient (p.767) | 1 |
| The French Physical Medicine and Rehabilitation Society (SOFMER)  2007 [66] | -- | -- | -- | -- | Perform a preoperative needs analysis (evaluation of patient needs), especially for the most fragile patients. A preoperative needs analysis can predict the kind of postoperative rehabilitation required and establish possible corrective measures during the preoperative period. The use of a predictive orientation questionnaire such as the Risk Assessment and Prediction Tool (RAPT) could allow for better preparation (p.196) | A preoperative rehabilitation program comprising at least physical therapy and education is recommended (p.196) | 2 |
| British Society for Rheumatology  2005 [67] | -- | -- | -- | -- | Exercise therapy for OA of the hip or knee should be individualized and patient-centred taking into account factors such as age, co-morbidity and overall mobility. Many factors such as the patient’s beliefs and experiences concerning OA, their expectations from their disease and its management, the requirements of the patient’s daily routine and their co-morbid disease and its treatment should be considered when prescribing exercise therapy, particularly as these have implications for adherence (p.69)  Group exercise and home exercise are equally effective and patient preference should be considered (p.70) | Both strengthening and aerobic exercise can reduce pain and improve function and health status in patients with knee and hip OA (p.69)  Prescription of both general (aerobic fitness training) and local (strengthening) exercises is an essential, core aspect of management for every patient with hip or knee OA (p.69)  To be effective, exercise programmes should include advice and education to promote a positive lifestyle change with an increase in physical activity (p.69)  Advice and education delivered in the form of self-efficacy enhancing information, a group-mediated cognitive behavioural intervention, an exercise dosage prescription instrument, individually tailored exercise prescription, and one-to-one person-centred exercise consultation leads to increased physical activity. Motivational interviewing has also been shown to increase physical activity but activity levels were not maintained at 1 yr (p.70)  Strategies to improve and maintain adherence should be adopted, e.g., long-term monitoring/ review and inclusion of spouse/family in exercise. In the general exercise literature, telephone contact, personal trainers and financial incentives have been shown to increase adoption of exercise programmes. Self-monitoring by means of a diary, reinforcement by another individual, telephone and mail contact, and graphic feedback increase maintenance. Married couples have been shown to attend an exercise programme more frequently if they attend with their spouse, and support from family and friends predicts maintenance of adherence (p.70) | 2 |
| European Alliance of Associations for Rheumatology (EULAR)  2005 [68] | -- | -- | -- | -- | Treatment of hip OA should be tailored according to: (a) Hip risk factors (obesity, adverse mechanical factors, physical activity, dysplasia) (b) General risk factors (age, sex, comorbidity, co-medication) (c) Level of pain intensity, disability, and handicap (d) Location and degree of structural damage (e) Wishes and expectations of the patient (p.672)  Any management plan requires consideration of patient beliefs and expectations and a holistic approach that takes into account comorbidity and other treatment requirements (p.672) | Non-pharmacological treatment of hip OA should include regular education, exercise, appliances (stick, insoles) and weight reduction if obese or overweight (p.673) | 2 |
| European Alliance of Associations for Rheumatology (EULAR)  2003 [69] | -- | -- | -- | -- | The treatment of knee OA should be tailored according to: -Knee risk factors (obesity, adverse mechanical factors, physical activity) -General risk factors (age, comorbidity, polypharmacy)  -Level of pain intensity and disability -Sign of inflammation for example, effusion -Location and degree of structural damage (p.1148)  Practitioners should tailor any treatment to the individual needs of the patient and this concept can be discussed within education (p.1148)  Each health professional must decide with each patient the most appropriate management plan at a particular time and for that location. Many patient-centred factors are important in determining the selection of treatments for individual patients with knee OA—for example, psychosocial factors and OA status; comorbid disease and drugs; patient beliefs about their knee OA; patient beliefs and preferences for its management; and previous patient experiences of treatments and health professionals (p.1154)  The management plan for patients with knee OA has to be individualised, reviewed, and adjusted in the light of the patient’s response and adherence and will vary between patients and between locations (p.1154) | Exercise programme, physiotherapy, weight loss combined with exercise, education, and wedged insoles, offer additional benefit when used with an analgesic or NSAID regimen (p.1148)  Non-pharmacological treatment of knee OA should include, education, exercise, appliances (sticks, insoles, knee bracing) and weight reduction (p.1148)  Education techniques shown to be effective include individualised education packages, regular telephone calls, group education, patient coping skills, and spouse assisted coping skills training (p.1149) | 2 |
