# Supplementary material for: Identifying strategies that support equitable person-centred osteoarthritis care for diverse women: content analysis of guidelines
Source: BMC Musculoskelet Disord. 2023 Sep 14;24:734. doi: 10.1186/s12891-023-06877-x (PMC10500823; doi:10.1186/s12891-023-06877-x)
Supplement: Supplementary file 5 — Additional File 5. Data extracted from included guidelines on strategies to achieve equitable access to OA care [file 12891_2023_6877_MOESM5_ESM.docx]

**Additional File 5. Data extracted from included guidelines on strategies to achieve equitable access to OA care**

| Guideline Year [Reference] | Prevalence of OA by intersectional factors | Barriers or challenges among vulnerable groups | Strategies needed or recommended to improve equitable access to and quality of care for disadvantaged groups | Domains addressed  (n) |
| --- | --- | --- | --- | --- |
| American Academy of Orthopedic Surgeons  2021 [9] | Worldwide prevalence of radiographically confirmed symptomatic knee OA is estimated to be 3.8% overall, increasing with age to over 10% in the population over the age of 60 (p.16)  Risk factors of [osteoarthritis] increase with age, especially in women. Although women represent 51% of the general population in the United States, they represent 78% of the patients diagnosed with osteoarthritis between 2008 and 2014 (p.16) | Since [topical NSAIDs] will now be available over the counter, prescription coverage will be less. This could pose as a barrier to those without health savings accounts or without insurance (p.31)  Most exercise programs would be considered feasible. However, some patients may have difficulty with access to supervised exercise due to travel or co-pay concerns. Aquatic programs would not be feasible for patients who do not have access to a pool or walking tank (p.33)  Neuromuscular exercise programs would be considered feasible. However, some patients may have difficulty with access to supervised exercise due to travel or co-pay concerns (p.34)  Self-management programs are feasible for patients provided they have appropriate access. Some patients may have limited access for participation, making the programs less feasible (p.35)  The manual therapy interventions are feasible for patients who have access to in-person physical therapy (p.41)  [Massage] interventions are considered feasible and relatively accessible to the general public. Patients who have transportation issues may have difficulty with obtaining access to treatment (p.42)  [Percutaneous Electrical Nerve Stimulation] is feasible but requires a practitioner trained in the technique which could limit access for some patients. [Pulsed Electromagnetic Field Therapy] is not widely used in clinics treating patients for knee osteoarthritis and thus could limit access for some patients (p.48)  [Extracorporeal Shockwave Therapy] is considered feasible but may not be widely available in clinics managing patients with knee osteoarthritis and therefore may not be accessible for all patients (p.49) | -- | 2 |
| American College of Rheumatology/Arthritis Foundation  2020 [10] | -- | -- | PATIENT  Self-management advice (tailored)  Exercise recommendations to patients should focus on the patient’s preferences and access, both of which may be important barriers to participation. If a patient does not find a certain form of exercise acceptable or cannot afford to participate or arrange transportation to participate, he or she is not likely to get any benefit from the suggestion to pursue that exercise … the availability, accessibility, and affordability of some [educational, physical, behavioral, psychosocial, mind-body, and pharmacologic] interventions vary, but in many communities the Arthritis Foundation, as well as local hospitals and other health-related agencies, offer free self-efficacy and self-management programs (p.154,160) | 1 |
| American Physical Therapy Association  2020 [36] | In 2010, the prevalence of knee osteoarthritis in North America was 3.1%, and globally the prevalence was 3.8%. Prevalence was higher in women and peaked at around 50 years of age (p.1604) | -- | -- | 1 |
| American Academy of Orthopaedic Surgeons  2020 [37] | Glenohumeral joint osteoarthritis is more common in women and increases with age. Primary glenohumeral joint osteoarthritis can occur over a broad age range, it is most commonly seen in patients >60 years of age. Radiographic data has found a prevalence rate of 94% in women and 85% in men over the age of 80 years (p.15) | -- | -- | 1 |
| Rheumatology and Immunology Expert Committee of the Cross-Strait Medical and Health Exchange Association  2020 [38] | The incidence of OA increased significantly with age (4), 10–17% in the population over 40 years old, 50% in the population over 60 years old, and 80% in the population over 75 years old, and the disability rate was 53% (p.2)  The incidence of OA is higher in females than in males, and higher in rural areas than in urban areas (p.2) | -- | -- | 1 |
| American Academy of Orthopaedic Surgeons  2020 [39] | -- | -- | -- | 0 |
| The Italian Society for Rheumatology  2019 [40] | -- | -- | -- | 0 |
| Osteoarthritis Research Society International  2019 [41] | -- | Aquatic exercise, though it is supported by a modest evidence base and demonstrates robust benefits on pain and objective measures of function, received a conditional recommendation because of accessibility issues, financial burden, as well as issues with uptake (p.1583) | -- | 1 |
| European Society for Clinical and Economic Aspects of Osteoporosis, Osteoarthritis and Musculoskeletal Diseases (ESCEO)  2019 [42] | Knee OA is the most common OA localization, and symptomatic knee OA is highly prevalent among people aged over 50 years…OA is a leading cause of pain in older people, and pain of the hip and knee results in physical disability and an increased risk of all-cause mortality (p.338) | -- | -- | 1 |
| European Alliance of Associations for Rheumatology (EULAR)  2019 [43] | Hand osteoarthritis (OA) is a common musculoskeletal disease, with prevalence rising steeply with increasing age (p.16) | -- | -- | 1 |
| European Alliance of Associations for Rheumatology (EULAR)  2018 [44] | -- | -- | -- | 0 |
| European Alliance of Associations for Rheumatology (EULAR)  2018 [45] | -- | -- | -- | 0 |
| The Ottawa Panel  2017 [46] | -- | -- | -- | 0 |
| The Ottawa Panel  2017 [47] | -- | Any type of strengthening exercise among the included trials on land-based exercise was identified as effective in this systematic review. However, some strengthening exercises, such the ones that require isokinetic resistance devices, have accessibility barriers for healthcare professionals and patients at the clinic or home settings (p.607) | -- | 1 |
| The Ottawa Panel  2017 [48] | -- | -- | -- | 0 |
| American Physical Therapy Association  2017 [49] | Prevalence [for hip and knee OA] was higher for females than males (p.7)  Osteoarthritis is the most common cause of hip pain in older adults (older than 50 years of age) (p.7)  Living in a community with a high poverty level is independently associated with radiographic OA in 1 or both hips. Low education attainment is independently associated with symptomatic OA of 1 or both hips (p.8)  Age, history of hip developmental disorders, previous hip joint injury, reduced hip ROM (especially hip IR), presence of osteophytes, lower socioeconomic status, higher bone mass, and higher BMI are risk factors for developing hip OA (p.8) | -- | -- | 1 |
| European Alliance of Associations for Rheumatology (EULAR)  2017 [50] | -- | -- | -- | 0 |
| Turkish League Against Rheumatism (TLAR)  2017 [51] | -- | -- | -- | 0 |
| Pan-American League of Associations for Rheumatology (PANLAR)  2016 [52] | Multiple patient factors are associated with an increased risk of OA, with age being the most important, followed by gender, body mass index, and microtraumas (p.346) | -- | -- | 1 |
| The Ottawa Panel  2016 [53] | Generally, there is a greater prevalence of osteoarthritis among women than men (p.936) | -- | -- | 1 |
| American Academy of Orthopaedic Surgeons  2015 [54] | -- | -- | -- | 0 |
| European Alliance of Associations for Rheumatology (EULAR)  2013 [55] | In elderly people, OA is the most common cause of disability, including pain and limitations of activities and participation (p.1125) | -- | -- | 1 |
| The Ottawa Panel  2012 [56] | Incidence is higher among women after the age of 55 (p.1270)  The majority of older adults will develop OA in 1 or several joints (p.1270) | -- | -- | 1 |
| Pan-American League of Associations for Rheumatology (PANLAR)  2011 [57] | -- | -- | -- | 0 |
| The Ottawa Panel  2011 [58] | -- | -- | -- | 0 |
| The Ottawa Panel  2011 [59] | People of all ages are susceptible to develop OA, but as people age the incidence rate increases. [OA] prevalence is higher in women after the age of 55 (p.320) | -- | -- | 1 |
| European Alliance of Associations for Rheumatology (EULAR)  2010 [60] | Risk factors that are strongly associated with the incidence of knee OA can help to identify patients in whom knee OA is the most likely diagnosis. These include increasing age over 50 years, female gender, higher body mass index, previous knee injury or malalignment, joint laxity, occupational or recreational usage, family history and the presence of Heberden’s nodes (p.485) | -- | -- | 1 |
| The French Physical Medicine and Rehabilitation Society (SOFMER)  2009 [61] | -- | -- | -- | 0 |
| The French Physical Medicine and Rehabilitation Society (SOFMER)  2008 [62] | -- | -- | -- | 0 |
| The French Physical Medicine and Rehabilitation Society (SOFMER)  2008 [63] | -- | -- | -- | 0 |
| The French Physical Medicine and Rehabilitation Society (SOFMER)  2007 [64] | -- | -- | -- | 0 |
| The French Physical Medicine and Rehabilitation Society (SOFMER)  2007 [65] | -- | -- | -- | 0 |
| The French Physical Medicine and Rehabilitation Society (SOFMER)  2007 [66] | -- | -- | CLINICIAN  Clinical assessment tools (tailored)  Rehabilitation requires a preoperative evaluation of patient needs and referral to a qualified health care professional in the patient’s education and preparation for the return home… Recommendation to perform preoperative needs analysis of patients [is suggested, especially for] a more fragile population in terms of difficulties in returning home, presurgical major disability, or precarious social conditions or comorbidities…the use of a predictive orientation questionnaire such as the Risk Assessment and Predictor Tool could allow for better preparation (p.196) | 1 |
| British Society for Rheumatology  2005 [67] | -- | -- | -- | 0 |
| European Alliance of Associations for Rheumatology (EULAR)  2005 [68] | -- | -- | -- | 0 |
| European Alliance of Associations for Rheumatology (EULAR)  2003 [69] | OA of the knee, the principal large joint to be affected, results in disabling knee symptoms in an estimated 10% of people older than 55 years, a quarter of whom are severely disabled. The risk of disability attributable to knee OA alone is as great as that due to cardiac disease and greater than that due to any other medical disorder in the elderly (p.1145)  A recent World Health Organization report on the global burden of disease indicates that knee OA is likely to become the fourth most important global cause of disability in women and the eighth most important in men (p.1145) | -- | -- | 1 |
